# Indolenine-substituted pyrazole derivative 4e inhibits planktonic *Staphylococcus lugdunensis* growth and biofilm formation by disrupting purine biosynthesis and compromising cell wall and membrane integrity

**DOI:** 10.1128/aac.00199-25

**Published:** 2025-07-23

**Authors:** Jazon Harl Hidrosollo, Hsiao-Wei Liao, Cheng Hong Yap, Jason Jonah James, Jang-Jih Lu, Yu-Hsien Tai, Chuancheng Wei, Tran Thi Dieu Thuy, Sek Peng Chin, Sun Tee Tay, Chin Fei Chee, Cheng Yen Kao

**Affiliations:** 1 Institute of Microbiology and Immunology, College of Life Sciences, National Yang Ming Chiao Tung University 34914, Taipei city, Taiwan; 2 College of Pharmacy and Medical Technology, University of San Agustin 63149, Iloilo, Philippines; 3 Department of Pharmacy, College of Pharmaceutical Sciences, National Yang Ming Chiao Tung University 390197, Taipei city, Taiwan; 4 Department of Medical Microbiology, Faculty of Medicine, Universiti Malaya 595424 , Kuala Lumpur, Malaysia; 5 Nanotechnology and Catalysis Research Centre, Universiti Malaya 595336 , Kuala Lumpur, Malaysia; 6 Division of Clinical Pathology, Taipei Tzu Chi Hospital, Buddhist Tzu Chi Medical Foundation 505876, New Taipei city, Taiwan; 7 Department of Pharmaceutical Chemistry, Faculty of Pharmacy, Universiti Malaya 595333 , Kuala Lumpur, Malaysia; 8 Health Innovation Center, National Yang Ming Chiao Tung University 34914, Taipei city, Taiwan; 9 Microbiota Research Center, National Yang Ming Chiao Tung University 34914, Taipei city, Taiwan; University of California San Francisco, San Francisco, California, USA

**Keywords:** antibacterial activity, biofilm inhibition, biofilm dispersal agent, indolenine-substituted pyrazole derivative, oxacillin-resistant *Staphylococcus lugdunensis*, untargeted metabolomics

## Abstract

*Staphylococcus lugdunensis* is an emerging nosocomial pathogen responsible for biofilm-related infections. Here, we explored the antibacterial and antibiofilm properties of the novel indolenine derivative 4e against *S. lugdunensis* and investigated its mechanisms of action. Its antibacterial and antibiofilm activities were assessed against oxacillin-resistant *S. lugdunensis* CGMH-SL131 using *in vitro* and *in vivo* models, including human cell lines, *Galleria mellonella* larvae, and mice. Mechanistic insights were explored via untargeted metabolomics. 4e exhibited bacteriostatic activity against a panel of gram-positive bacteria, with a 1× minimum inhibitory concentration (MIC) of 62.5 µg/mL. Scanning electron microscope observations of cells treated with 0.5% SDS and 1× MIC 4e displayed signs of cell shape distortion, including complete shrinkage and bursting. 4e effectively inhibited biofilm formation by 54.3% at 1.56 µg/mL, and the minimum biofilm inhibition concentration 80% (MBIC_80_) was 3.125 µg/mL. In addition, 70.3% of 1-day preformed biofilms were dispersed at 1× MBIC_80_. 4e exhibited low cytotoxicity (>85% survival) in HaCaT, H10975, and Caco-2 cells at 1× MIC. When administered 1 hour post-infection, 4e (3.125 mg/kg) improved larval survival to 90%, matching tigecycline (2 mg/kg), whereas untreated larvae had only 20% survival after 7 days. In C57BL/6 mice, 4e (2.5 mg/kg) reduced kidney bacterial loads from 10⁷ to 5.3 × 10⁴ CFU. Untargeted metabolomics suggests that 4e’s antibacterial and antibiofilm effects result from disrupting purine biosynthesis and compromising cell wall and membrane integrity. These findings highlight 4e as a promising new antibiofilm agent and potential alternative treatment for biofilm-related infections caused by *S. lugdunensis* and multidrug-resistant *Staphylococcus* species.

## INTRODUCTION

*Staphylococcus lugdunensis* belongs to coagulase-negative staphylococci (CoNS), which colonize moist areas of the human skin and nostrils like normal flora (1, 2). Recently, *S. lugdunensis* was discovered to be a lugdunin-producing bacterium that was notably studied for its broad inhibitory activities against pathogenic gram-positive bacteria (3). However, *S. lugdunensis* could also cause self-limiting to life-threatening infections since it shares several characteristics with *S. aureus*, a highly invasive opportunistic pathogen (4). *S. lugdunensis* can infect highly susceptible hosts, including immunocompromised individuals, humans with wounds or skin injury, and hospitalized patients who may have direct contact with contaminated indwelling medical devices, causing catheter-related bacteremia (5, 6). In severe cases, *S. lugdunensis* was emphasized as the most virulent pathogen among the CoNS, since it causes infective endocarditis, which is a disease exhibiting aggressive heart valve destruction leading to high mortality rates worldwide (7–9).

Biofilm formation is the most common virulence factor of medically important pathogens, enabling the bacteria to colonize host tissues and persist after the initial infection (10). These multicellular bacterial communities are held together by a matrix composed of biological materials, such as polysaccharide intercellular adhesion, extracellular DNA (eDNA), and proteinaceous factors (11, 12). The extracellular matrix acts as a barrier, preventing antibiotics from effectively penetrating and reaching the bacteria (13, 14). This enhanced resistance significantly complicates treatment options for infections associated with biofilm-forming bacteria (14).

Recently, we reported a 12-year longitudinal epidemiological study to characterize *S. lugdunensis* isolated from sterile body fluids in a single medical center in Taiwan (15). Our findings revealed that 138 out of 438 (33.3%) *S*. *lugdunensis* isolates were resistant to oxacillin. In addition, oxacillin-resistant *S. lugdunensis* (ORSL) exhibited high resistance rates to clindamycin (43.2%), erythromycin (43.2%), gentamicin (78.1%), and tetracycline (46.6%). Therefore, developing novel antibacterial or antibiofilm agents targeting bacteremia ORSL strains has become an urgent priority in drug development.

The application of synthetic organic chemistry to generate small-molecule drugs and their derivatives has become a promising strategy to search for new bioactive agents for infectious diseases (16). Indole is a multifaceted pharmacophore and a privileged scaffold, recognized as an exceptional heterocyclic compound with diverse pharmacological properties (17). Synthetic indole derivatives showed remarkable antimicrobial and antibiofilm activity against various *S. aureus* and *Escherichia coli* strains (18, 19). The inhibitory effect of 2-aryl-5-nitro-1*H*-indoles against the NorA efflux pump of *S. aureus*, which is responsible for multidrug resistance, was thoroughly investigated in a previous study (20). This makes indole derivatives an excellent drug lead for overcoming drug resistance (18). In addition, a class of 2-(6-phenylimidazo[2,-1-b][1,3,4]thiadiazol-2-yl)−1*H*-indoles was efficiently synthesized and was observed to exhibit antibiofilm activities against the gram-positive bacterial reference strains (19). Considering the reports on indole targets in the past decades, combining the promising scaffolds would be beneficial in developing new therapeutic agents. In our previous work, we demonstrated the efficacy of 4e as a potent biofilm inhibitor from our small library of indolenine-substituted pyrazoles, against both methicillin-susceptible *S. aureus* (MSSA) ATCC 29213 and methicillin-resistant *S. aureus* (MRSA) ATCC 33591 (21).

In this current study, we further explored the dose-dependent *in vitro* and *in vivo* antibacterial, antibiofilm, and cytotoxicity properties of 4e against a member of CoNS. We used a bacteremia isolate, CGMH-SL131, which was obtained from a catheter-related infection. This strain exhibits high-level resistance to oxacillin, robust biofilm-forming capability, and has publicly available whole-genome sequence data (NCBI GenBank accession number: NZ_CP048007.1). In contrast to our previous work, this current study presents, for the first time, the low cytotoxic effects of 4e on the immortalized keratinocyte cell line (HaCaT), human embryonic kidney cell line (HEK293), lung (H10975) and colon (Caco-2) adenocarcinoma cell lines, *in vivo* efficacy in *G. mellonella* larvae, and C57BL/6 mice infection models. Lastly, the potential mechanism of action on 4e-treated planktonic cells was explored using untargeted metabolomics.

## RESULTS

### 4e exhibits bacteriostatic activity on CGMH-SL131

**Fig 1:**
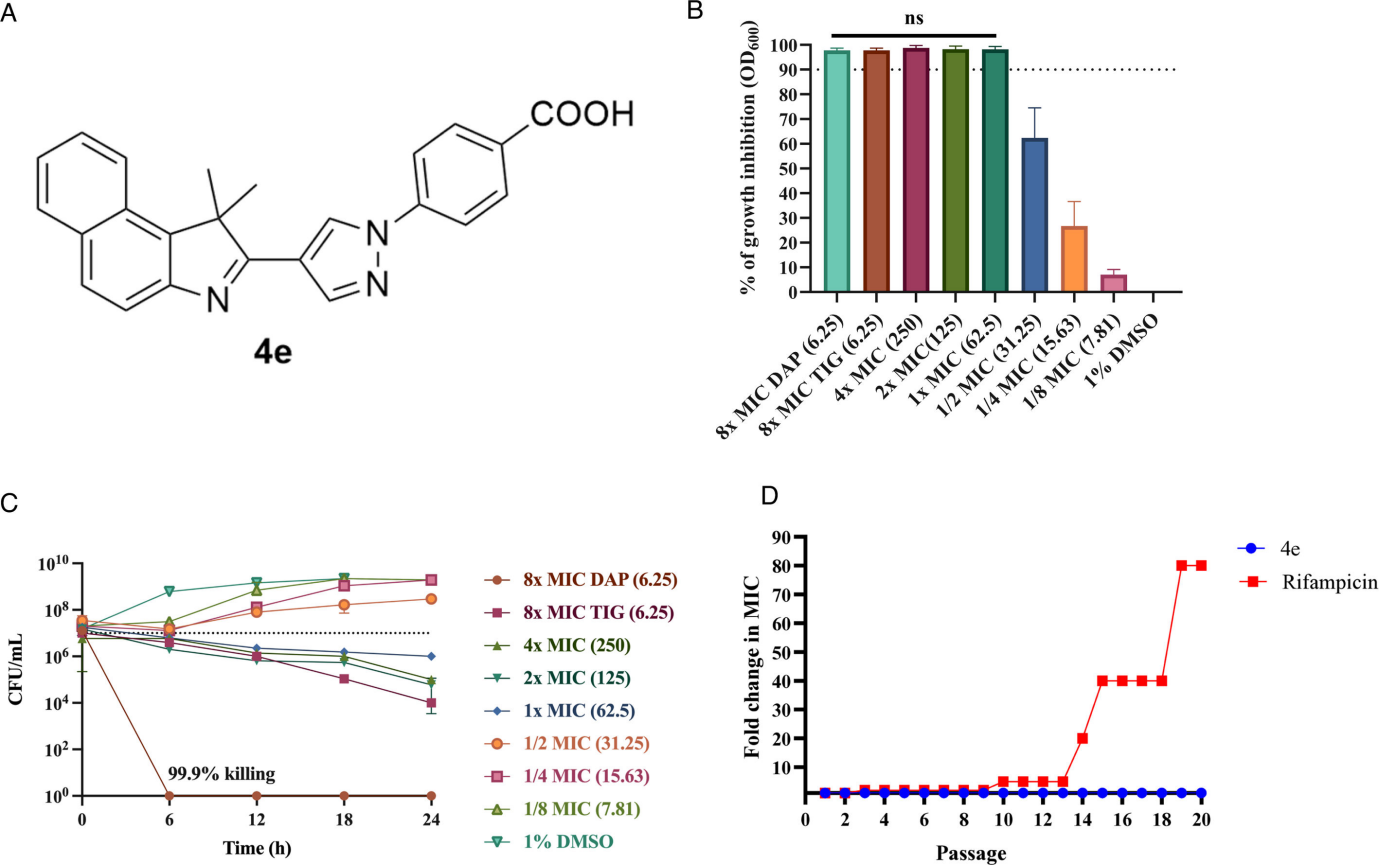
Antibacterial activity of 4e against *Staphylococcus lugdunensis* CGMH-SL131. (**A**) 4e chemical structure. (**B**) Cultures of CGMH-SL131 (1 × 10^7^ CFU/mL) were treated with a twofold serial dilution of 4e to determine bacterial growth inhibition by broth microdilution. The percentage of growth inhibition was calculated based on the OD_600_ at the 24 h endpoint. TIG (tigecycline, 8x MIC) and DAP (daptomycin, 8x MIC) were used as positive controls, and 1% DMSO as growth control. The dashed line represents 90% inhibition of growth, ns = not significant (*P* > 0.05). (**C**) Bacterial colony count (CFU/mL) was calculated at a 6 h interval from time 0 until 24 h, and surviving colonies were plated out in TSA for enumeration. MIC of 4e was defined as the lowest concentration with at least ≥90% growth inhibition and with no broth turbidity visible to the naked eye. The concentration is indicated in parentheses (μg/mL). All experiments were done in biological and technical triplicate. (**D**) Multi-passage resistance of 4e was assessed for 20 days. The experiment was done in one biological and three technical replicates.

4e is a newly synthesized indolenine-substituted pyrazole compound containing a fused benzene indolenine moiety and a benzoic acid linked to the N1 position of the pyrazole nucleus (Fig. 1A). To elucidate its antibacterial activity, a twofold microbroth dilution of 4e (250 μg/mL–1.95 μg/mL) was used in a 96-well plate assay. The percentage of growth inhibition was calculated after 24 h of incubation. 1× minimum inhibitory concentration (MIC) was determined based on the lowest concentration required to prevent turbidity in broth visible by the naked eye, with at least ≥90% growth inhibition. Our results demonstrated that concentrations from 1× MIC (62.5 µg/mL) to 4× MIC (250 µg/mL) resulted in 90% growth inhibition, which is comparable to the antibacterial activities of tigecycline and daptomycin at 8× MIC (Fig. 1B). The 4e concentrations of 31.25 µg/mL, 15.63 µg/mL, and 7.81 µg/mL individually inhibited bacterial growth by 62.4%, 26.7%, and 7.1%, respectively. Importantly, time-dependent killing kinetics revealed that 1× MIC of 4e elicited bacteriostatic activity as indicated by the significant 3-log reduction in colony-forming units (CFUs)/mL when compared to 1% DMSO growth control (Fig. 1C). Bacteriostatic activity was also observed on tigecycline control. Only daptomycin had bactericidal activity, as shown by no surviving colonies (99.9% killing) at the 6 h timepoint (Fig. 1C).

In addition, when 4e was tested against another 21 clinical isolates of *S. lugdunensis*, only isolate CGMH-SL19 exhibited an MIC of 31.25 µg/mL (Table S2). The remaining 20 isolates, similar to CGMH-SL131, showed a MIC of 62.5 µg/mL, regardless of their susceptibility to oxacillin and other antibiotics, SCC*mec* types, or MLST.

Furthermore, 4e demonstrated activity against other gram-positive bacteria, including multidrug-resistant strains of *S. aureus*, vancomycin-intermediate-resistant *S. aureus* (VISA), heterogeneous VISA (hVISA), vancomycin-resistant *Enterococcus faecium*, and other clinical isolates such as *Staphylococcus saprophyticus*, *Staphylococcus epidermidis*, *Streptococcus agalactiae*, *Enterococcus faecalis*, *Enterococcus raffinosus,* and *Staphylococcus haemolyticus* (MIC values ranging from 31.25 to 125 µg/mL) (Table 1). However, gram-negative bacteria such as *Pseudomonas aeruginosa*, *Acinetobacter baumannii*, *Klebsiella pneumoniae*, and *E. coli* exhibited MIC values exceeding 125 µg/mL. Moreover, to assess the potential of 4e to induce drug resistance, we measured MIC values during continuous culturing at sub-MIC for 20 passages. Our results showed that rifampicin exhibited a 40-fold increase in MIC while the MIC of 4e remained unchanged throughout the experiment (Fig. 1D). These results suggest that 4e has a lower potential to induce drug resistance in CGMH-SL131 compared to rifampicin.

**TABLE 1:** Antibacterial activity of 4e against other gram-positive and gram-negative pathogens^*c*^

Pathogen	MIC (μg/mL)^*a*^	
	4e	TIG
Gram-positive		
*Staphylococcus aureus* USA300 LAC (MRSA)	62.5	0.78
*Staphylococcus aureus* NTUH-SA1 (MRSA)	62.5	0.78
*Staphylococcus aureus* ATCC 25923 (MSSA)	31.25	0.78
*Staphylococcus aureus* ATCC 29213 (MSSA)	125	1.56
*Staphylococcus aureus* CGMH-SA1205 (VISA)	62.5	0.78
*Staphylococcus aureus* CGMH-SA2 (heterogeneous VISA, hVISA)	62.5	0.78
*Staphylococcus epidermidis* NTUH-01	62.5	0.78
*Staphylococcus saprophyticus* TVGH-94-2	62.5	0.78
*Streptococcus agalactiae* (Group B) TVGH-58-2	31.25	0.19
Vancomycin-resistant *Enterococcus faecium* NTUH-EF4	31.25	0.39
*Enterococcus faecalis* TVGH-24-2	125	1.56
*Enterococcus raffinosus* TVGH-56-2	62.5	0.39
*Staphylococcus haemolyticus* CGMH-SH53	31.25	1.56
*Staphylococcus lugdunensis* CGMH-SL49 (OSSL)	62.5	0.78
*Staphylococcus lugdunensis* CGMH-SL11 (OSSL)	62.5	0.78
*Staphylococcus lugdunensis* CGMH-SL131 (ORSL)	62.5	0.78
Gram-negative		
*Pseudomonas aeruginosa* NTUH-PA06	>125	^*b*^
*Acinetobacter baumannii* NCKH-AB9291	>125	1.56
*Klebsiella pneumoniae* NTUH-KP442	>125	1.56
*Escherichia coli* NTUH-EC447	>125	0.39

^*a*^ MICs were determined by broth microdilution.

^*b*^ Intrinsic resistance to TIG.

^*c*^ TIG, tigecycline; VISA, vancomycin-intermediate *Staphylococcus aureus;* MRSA, methicillin-resistant *Staphylococcus aureus;* MSSA, methicillin-sensitive *Staphylococcus aureus*; ORSL, oxacillin-resistant *Staphylococcus lugdunensis*; OSSL, oxacillin-susceptible *Staphylococcus lugdunensis*.

### 4e disrupts the cell membrane barrier function of CGMH-SL131

**Fig 2:**
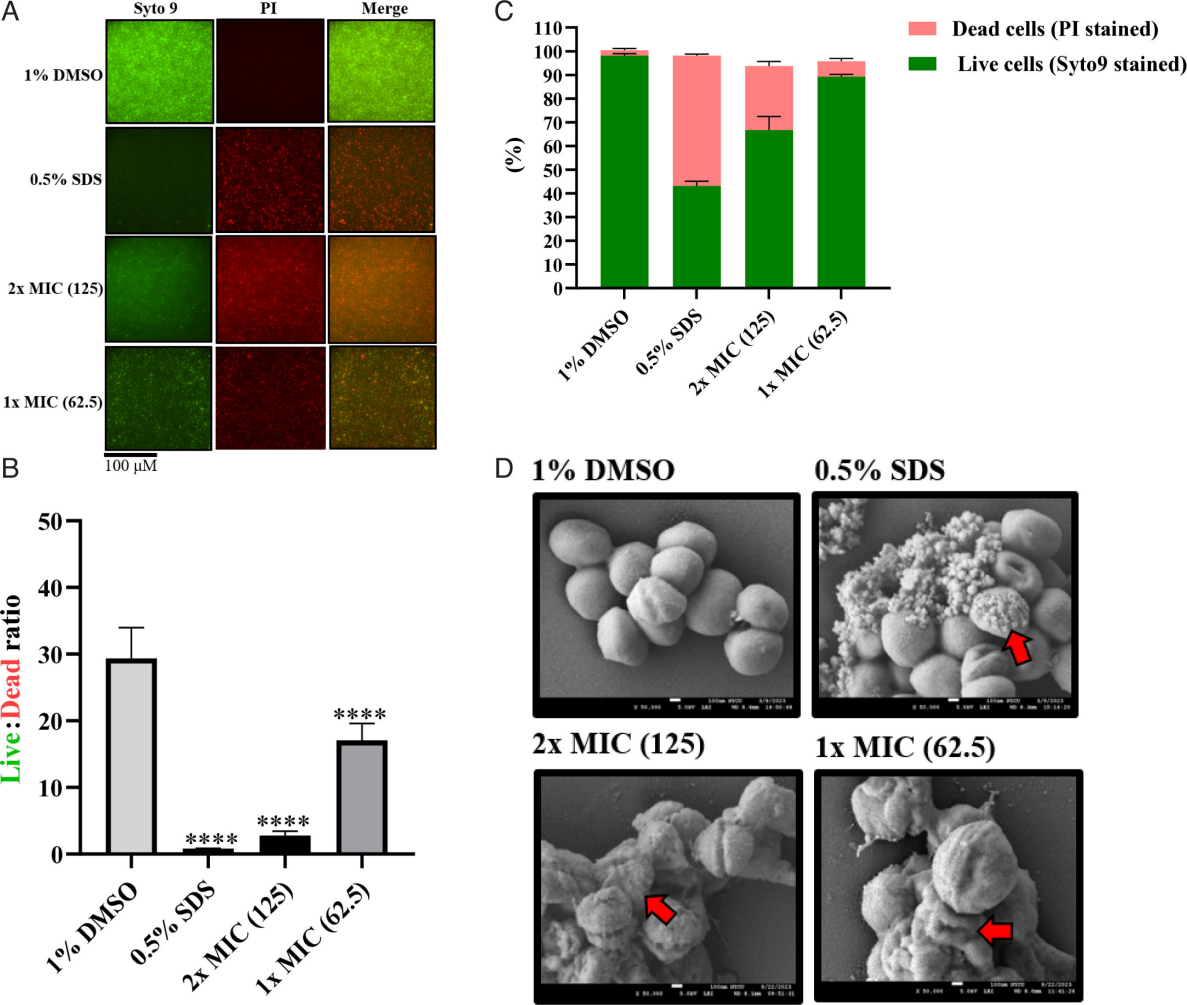
Cell membrane permeability effect of 4e to CGMH-SL131 using live (Syto9)/dead (PI) fluorescence and scanning electron microscopy. (**A**) Fluorescence images of CGMH-SL131 following viability staining on 4e-treated (1× MIC and 2× MIC) planktonic cells after 24 h using the IX83 Olympus inverted fluorescence microscope. 1% DMSO and 0.5% SDS were used as negative and positive controls, respectively. Cells stained with green fluorescence (Syto9) were viable, while those stained with red fluorescence (PI) were dead. (**B**) Live/dead ratio based on fluorescence intensity signals. (**C**) % live and dead cell population. All fluorescence assays were done in biological and technical triplicate, ****P < 0.0001. (**D**) Scanning electron micrographs of 4e-treated (1× MIC and 2× MIC) cells after 24 h using field emission SEM. The scale bar is at 100 µm. The concentration of 4e is indicated in parentheses (μg/mL). The red arrows indicate significant structural damage to *S. lugdunensis*. All images were representative of three independent biological trials with two technical replicates.

To further assess the damaging activity of 4e on cell membrane integrity, a fluorescence microscopic experiment, using fluorescent dyes, Syto9, and propidium iodide (PI), was performed. Syto9 is a cell-permeant dye that emits green fluorescence upon binding to nucleic acids of both live and dead bacterial cells. By contrast, PI emits red fluorescence that can only penetrate bacterial cells with compromised cell membrane integrity (dead cells). The fluorescence images of the cell population showed bacterial cell membranes became permeable to PI after 4e treatment at 1× MIC and 2× MIC after 24 h (Fig. 2A). The same PI-permeable cell population was observed on 0.5% SDS-treated bacterial cells (positive control) (Fig. 2A). The detection of both green and red fluorescence in the merged panel of the 4e-treated cells strongly supports its bacteriostatic activity (Fig. 2A). In addition, to generate a numerical estimation, the percentage of live and dead cells was calculated based on fluorescence intensity signals. In the 1% DMSO, a live:dead fluorescence ratio of approximately 33.3, corresponding to 98.3% viable cells, was observed (Fig. 2B and C). By contrast, 0.5% SDS-treated samples exhibited a live:dead fluorescence ratio of 0.82, with approximately 44.7% viable and 55.3% non-viable cells (Fig. 2B and C). In the 4e-treated group, 2× MIC revealed a live:dead fluorescence ratio of 2.8, wherein 73.3% were viable cells and 26.7% of the cell population were dead (Fig. 2B and C). Lastly, 1× MIC generated a live:dead fluorescence ratio of 17.0 with at least 89.7% of the cell population still viable (Fig. 2B and C). Furthermore, we used a potentiometric fluorophore, DiSC3(5), to assess membrane potential disruption with CCCP (5 µM) as a positive control. We noticed a drop in DiSC3(5) fluorescence in the 4e-treated cells (2× MIC and 1× MIC) when compared to the DMSO control group, and the membrane potential disruption is highly comparable with the CCCP-treated positive control (Fig. S1). These findings suggested that 4e exhibited growth inhibition by partially compromising cell membrane integrity, with a bacteriostatic effect with an impact on bacterial cell membrane potential.

### 4e severely damages the cell morphology of CGMH-SL131

The observed disruption of membrane barrier function by PI staining suggested 4e’s effect on cell structure. Therefore, we used SEM examination to observe CGMH-SL131 cell morphology after 4e treatment. The 1% DMSO group was used as a control to justify the observed differences in the 4e-treated cells. In the 1% DMSO-treated group, the cell shape was observed to exist as cocci colonies of grape shape, which is typical of *S. lugdunensis* shape (Fig. 2D). Interestingly, 50,000× magnification of SEM micrographs of 0.5% SDS and 4e (1× MIC and 2× MIC) treated cells displayed several signs of cell shape distortion, including completely shrunk and burst cells. In addition, cells resembling biconcave shape, sticky membranes, and deep craters were also observed (Fig. 2D). These findings highlighted the damaging effect of 4e on CGMH-SL131 cell structure.

### 4e has potent antibiofilm activity against CGMH-SL131

**Fig 3:**
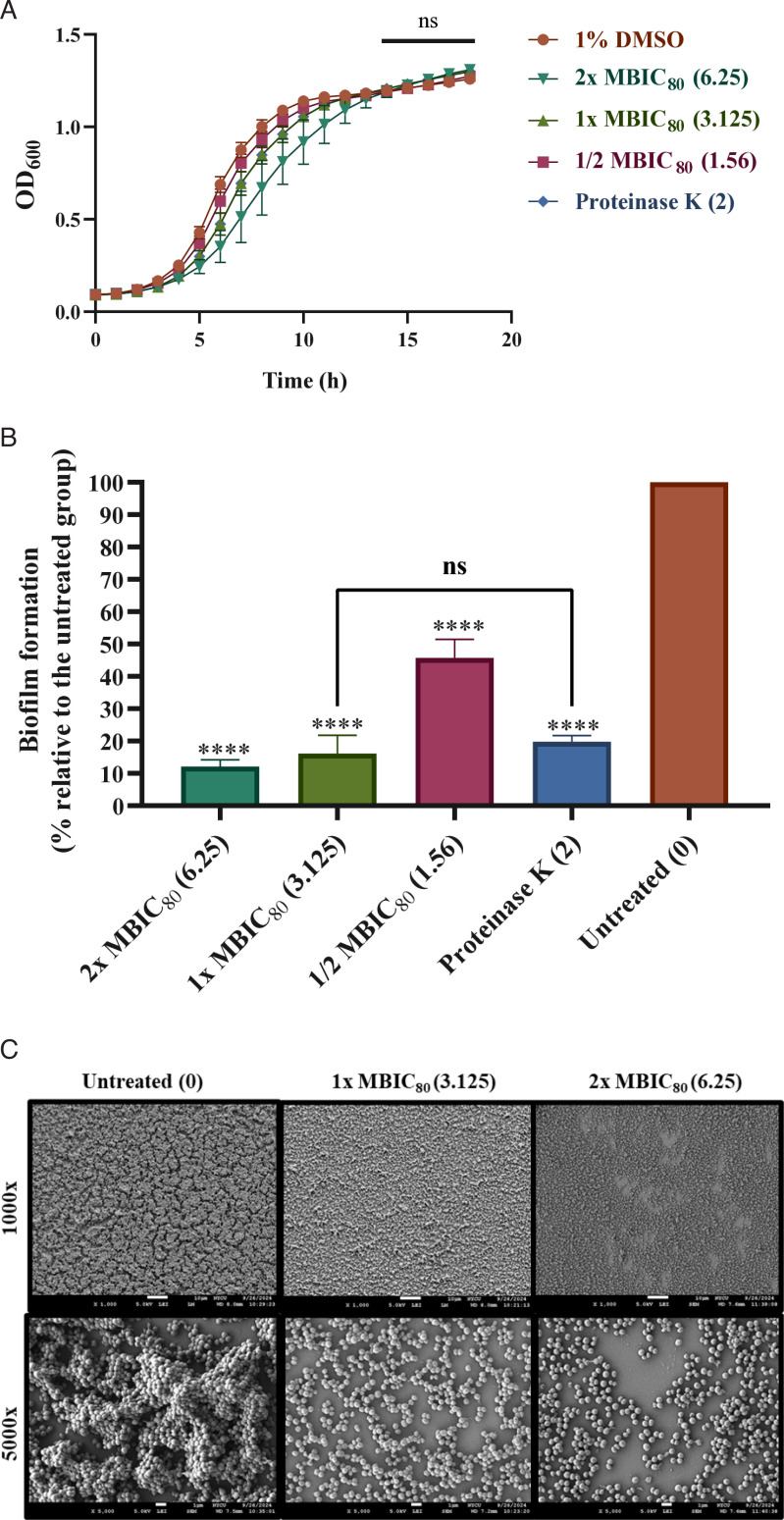
Biofilm inhibition effects of 4e against CGMH-SL131. (**A**) Growth curve. (**B**) Percentage of biofilm formation on 96-microplate wells treated with 2× MBIC_80_, 1× MBIC_80_, and 1/2 MBIC_80_ of 4e for 24 h. Cells with no 4e treatment were considered as the biofilm-positive control. All experiments were done in biological and technical triplicate, ****P < 0.0001, ns = not significant (*P* > 0.05). (**C**) Scanning electron micrographs of 4e-treated (2× MBIC_80_, 1× MBIC_80_) cells after 24 h using field emission SEM. The concentration of 4e is indicated in parentheses (μg/mL). The scale bar is at 10 µm. SEM experiments were done in three independent biological trials.

To evaluate the antibiofilm potential of compound 4e, we employed sub-inhibitory concentrations that exhibited no effect on the growth of planktonic cells. Biofilm formation was assessed under these conditions and compared to the positive control treatment with proteinase K (2  µg/mL) (Fig. 3A). It is noteworthy that 4e can inhibit biofilm formation at concentrations ranging from 1.56 to 6.25 μg/mL (Fig. 3B). The minimum biofilm inhibition concentration (MBIC_80_) was defined as the concentration that inhibits more than 80% of biofilm formation. It was shown that biofilm formation was inhibited by 54.3% at 1.56 µg/mL. Notably, 1× MBIC_80_ (3.125 µg/mL) and 2× MBIC_80_ (6.25 µg/mL) inhibited biofilm formation by 83.9% and 87.9%, respectively (Fig. 3B). In addition, the biofilm inhibition of 1× MBIC_80_ (3.125 µg/mL) was comparable to proteinase K. To visualize the biofilm architecture formed on the round glass slides after 4e treatment for 24 h, we used SEM to validate the biofilm inhibition activity. At 1,000× magnification, SEM analysis revealed a thick and fully established biofilm consisting of multi-layered bacterial cells (Fig. 3C). Under 1× MBIC_80_ treatment of 4e, biofilm formation was disrupted, and reduced biofilm mass was observed on the round glass slide (Fig. 3C). At 5,000× magnification, untreated cells appeared as large aggregates with overlapped layers (Fig. 3C). By contrast, cells treated with 1× MBIC_80_ presented reduced cell aggregation, and overlapped layers were negligible (Fig. 3C). An enhanced biofilm inhibition effect was noted upon supplementation with 2× MBIC_80_. Moreover, all 22 clinical isolates of *S. lugdunensis* were subjected to biofilm formation screening using 0.1% crystal violet staining, and we observed that all strains are strong biofilm-formers except for CGMH-SL33, which forms a moderate biofilm (Fig. S2). We next examined whether compound 4e retains its antibiofilm activity against a bacteremia isolate with a higher biofilm-forming capacity than CGMH-SL131. Therefore, the strain CGMH-SL11 exhibited a biofilm biomass (OD_595_) of 5.88, representing a 1.89-fold increase compared to CGMH-SL131 (Fig. S2) that was tested. Our results showed that 4e at lower concentrations consistently did not inhibit growth (Fig. S3A) but inhibited the biofilm formation at 1× MBIC_80_ (3.125 µg/mL) and 2× MBIC_80_ (6.25 µg/mL) by 86.7% and 86.4%, respectively, against CGMH-SL11 (Fig. S3B).

**Fig 4:**
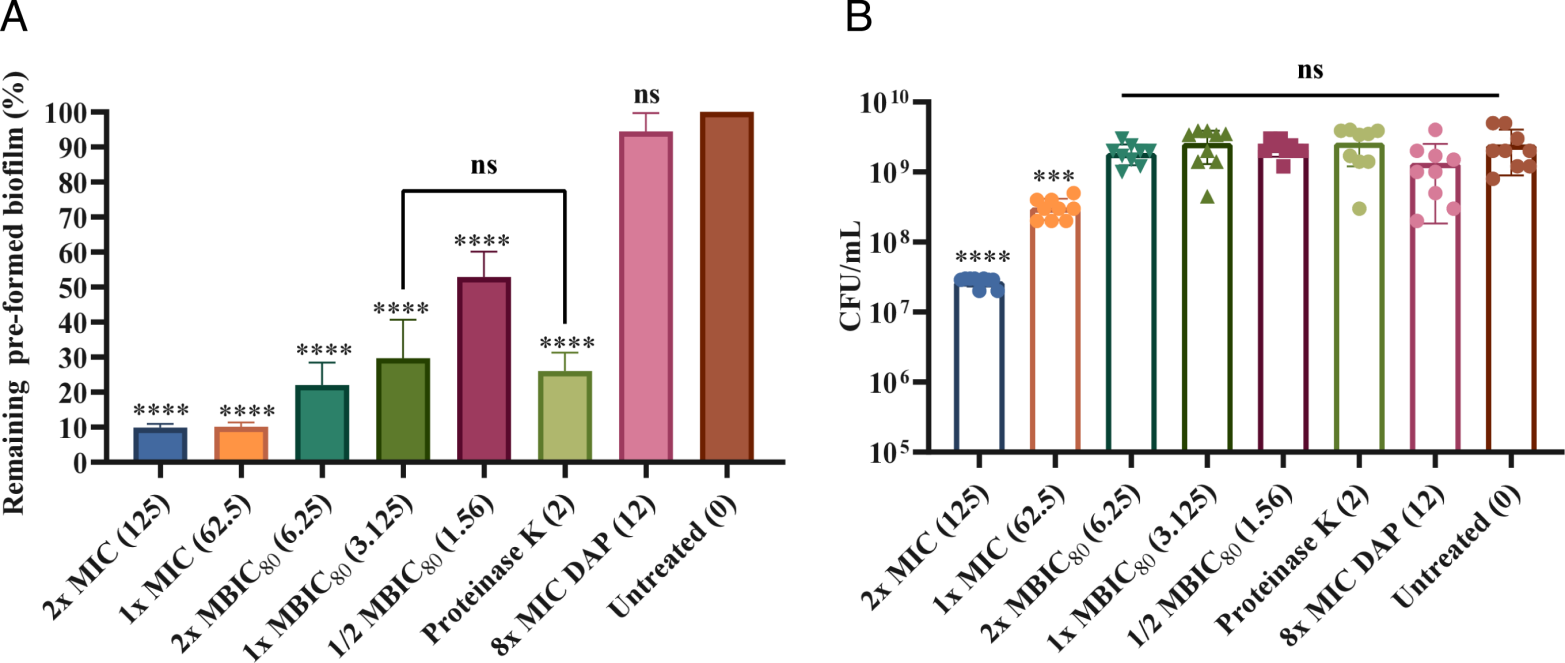
Biofilm dispersal and combined killing effects of 4e against CGMH-SL131 planktonic and biofilm cells. (**A**) Percentage of remaining preformed biofilm on 96-microplate wells treated with 2× MIC, 1× MIC, 2× MBIC_80_, 1× MBIC_80_, 1/2 MBIC_80,_ proteinase K, and 8× MIC daptomycin (DAP) for 24 h. (**B**) The CFU/mL represents the combined number of viable bacterial cells from both planktonic and biofilm populations after 24 h treatment with 2× MIC, 1× MIC, 2× MBIC_80_, 1× MBIC_80_, 1/2 MBIC_80,_ proteinase K, and 8× MIC DAP. The concentration of 4e and proteinase K is indicated in parentheses (μg/mL). All experiments were done in three biological and technical replicates. Statistical analysis was performed using the untreated group as the comparison control. ns= not significant (*P* > 0.05), **P < 0.01; ***P < 0.001; ****P < 0.0001.

The ability of 4e to inhibit biofilm formation prompted us to further investigate whether it can disperse 1-day-old biofilms. As it was previously described that *S. lugdunensis* biofilm matrix contains proteinaceous factors, proteinase K (2 µg/mL) was used as an antibiofilm dispersal control. We first allowed biofilms to form for 24 h and started 4e treatment for the next 24 h. In this experiment, we used 2× MIC, 1× MIC, 2× MBIC_80_, 1× MBIC_80_, 1/2 MBIC_80_, proteinase K (2 µg/mL), and daptomycin (8× MIC) as the treatment group and compared the results with the untreated group. Our results showed that all 4e-treated biofilm cells were significantly dispersed. Interestingly, 1× MIC and 2× MIC outperformed daptomycin in efficiently dispersing 1-day-old biofilms, with only about 10% of the preformed biofilm remaining for both 4e treatments observed (Fig. 4A). A dose-dependent dispersal effect was observed with 2× MBIC_80_ and 1× MBIC_80_, where 22.0% and 29.7% of biofilm cells remained, respectively (Fig. 4A). In addition, 47.1% of the biofilms were dispersed at 1/2 MBIC_80_ (Fig. 4A). Importantly, 4e at 1× MBIC_80_ is highly comparable with the biofilm dispersal activity of proteinase K (2 µg/mL) (Fig. 4A). Hence, the minimum biofilm dispersal concentration capable of dispersing mature biofilms by nearly 80% is 6.25 µg/mL. Notably, CFU counts representing the combined number of viable bacterial cells from both planktonic and biofilm populations after treatment also revealed a 2-log reduction under 2× MIC, a 1-log reduction under 1× MIC, and no significant CFU reduction was observed in daptomycin-treated biofilms (Fig. 4B).

Interestingly, once tested with CGMH-SL11, 4e concentration at 1× MBIC_80_ (3.125 µg/mL) and 1/2 MBIC_80_ (1.56 µg/mL) failed to disperse matured biofilms, as there was no significant difference compared to the untreated group (Fig. S3C). In addition, 2× MBIC_80_ was observed to weakly disperse the biofilms, as 61.8% of the biofilm still remained (Fig. S3C). However, it is still worth highlighting that 2× MIC, 1× MIC, and proteinase K consistently dispersed mature biofilms, where 22.0%, 14.8%, and 14.28% of biofilm cells remained, respectively (Fig. S3C). On the other hand, CFU counts representing the combined number of viable bacterial cells from both planktonic and biofilm populations after treatment also revealed a 3-log reduction under 2× MIC, a 1-log reduction under 1× MIC, and no significant CFU reduction was observed in daptomycin-treated biofilms (Fig. S3D). Taken together, these results further support the potential application of 4e as a potent biofilm inhibitor and dispersal agent with minimal biofilm cell killing at high concentrations.

### 4e exhibited low cytotoxicity to human-derived cell lines

**Fig 5:**
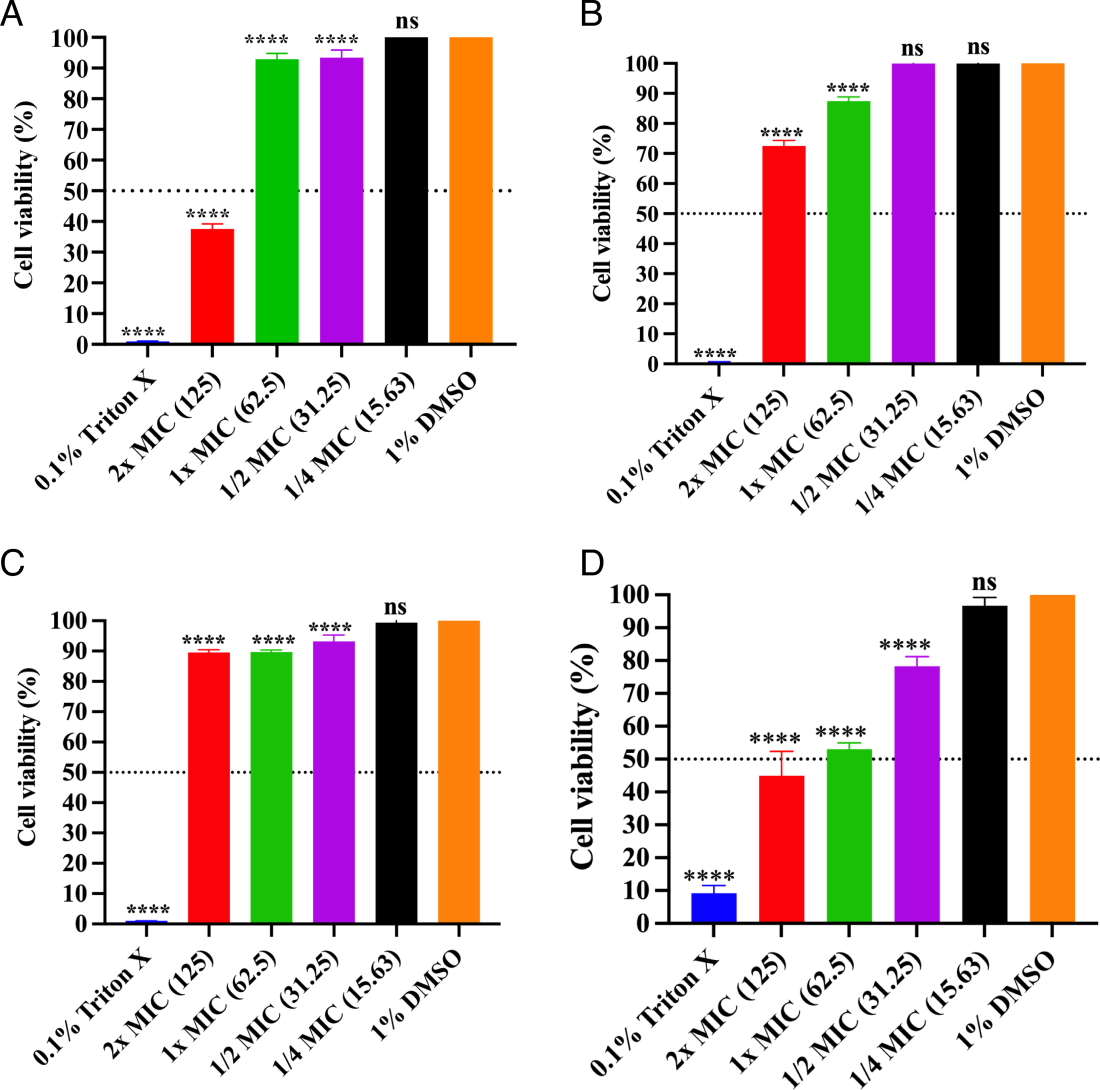
Cytotoxicity of 4e against human-derived cell lines using MTT assay. (**A**) H1975; lung adenocarcinoma. (**B**) HaCaT; immortalized keratinocytes. (**C**) Caco-2; colon adenocarcinoma. (**D**) HEK293; human embryonic kidney cells. All cell lines were exposed to varying concentrations of 4e for 24 h. 0.1% Triton X-100 and 1% DMSO were used as the toxic and non-toxic controls, respectively. The concentration of 4e is indicated in parentheses (μg/mL). The dashed line represents 50% survival of cells. All experiments were done in three biological and three technical replicates. Statistical analysis was performed using the 1% DMSO-treated group as the comparison control. ns= not significant (*P* > 0.05), ****P < 0.0001.

Cytotoxicity of 4e was evaluated against cell lines HaCaT, H1975, Caco-2, and HEK293 using the MTT assay. The half maximal inhibitory concentration (IC_50_) was observed to be ≥125 µg/mL (2× MIC) in all cell lines tested except for HEK293, of which IC_50_ was noted to be ≥62.5 µg/mL (1× MIC), indicating that the active concentration of 4e responsible for antibacterial activity (1× MIC) was significantly less toxic than 0.1% Triton X-100 control (Fig. 5). It was shown that 1× MIC of 4e treatment exhibited more or less 90% cell viability in HaCaT, H1975, and Caco-2 cell lines and 50% cell viability in HEK293. Importantly, no significant difference in cell viability was observed between 1/4 MIC and 1% DMSO control when tested in all cell lines (Fig. 5). On the other hand, both 1/2 MIC and 1/4 MIC were observed to be non-toxic to HaCaT cell lines (Fig. 5B). As mentioned, 4e is potent to inhibit biofilm formation at 3.125 µg/mL (1× MBIC_80_), which is 10-fold lower than the 1/2 MIC (31.25 µg/mL). Therefore, it is important to highlight that the active concentrations responsible for antibiofilm activity do not induce cytotoxicity. These findings support the potential future development of 4e-based antibiofilm products.

### 4e rescued the survival of *G. mellonella* larvae infected with CGMH-SL131

**Fig 6:**
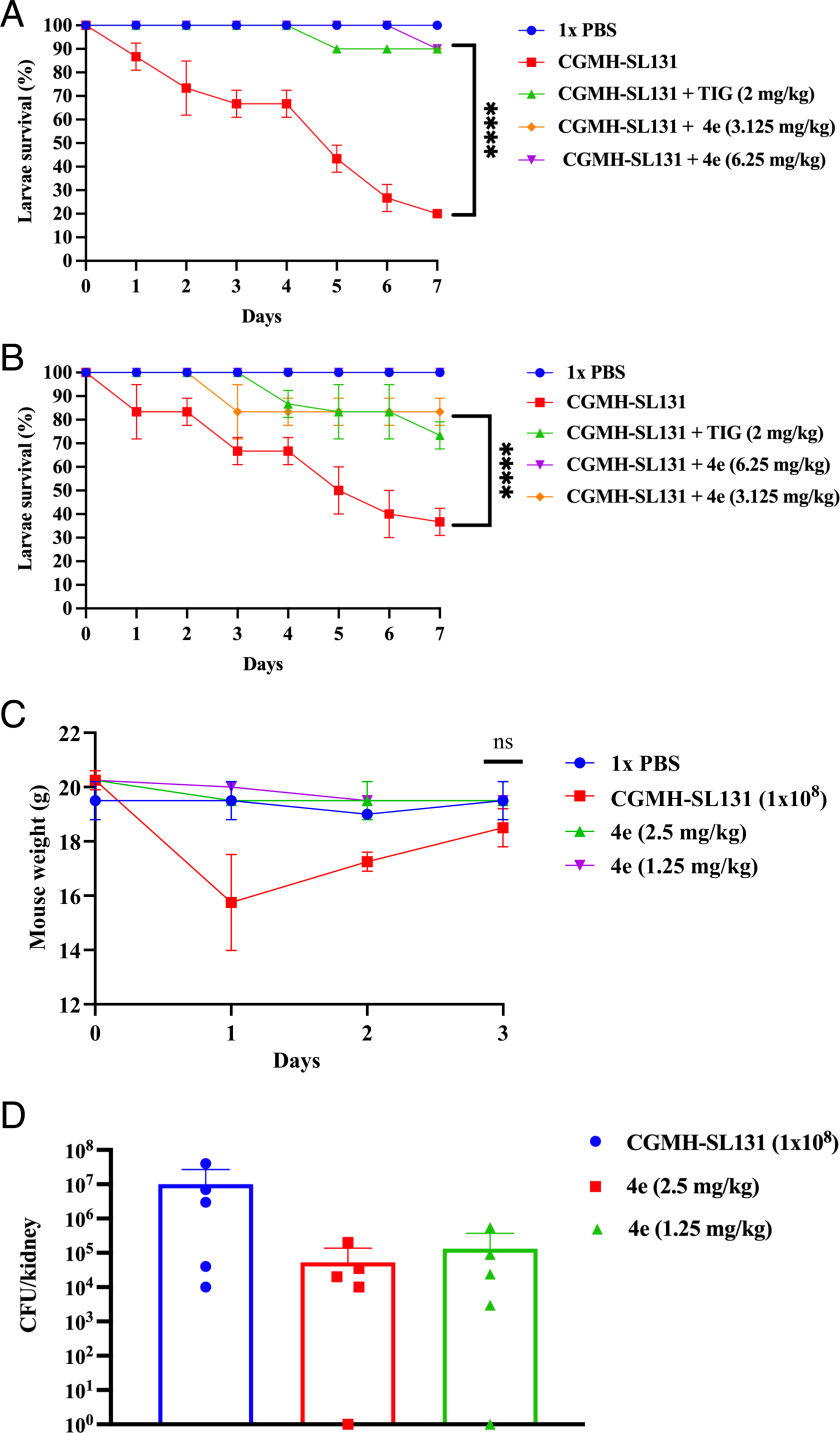
*G. mellonella* larvae and mice infection model. (**A**) 1 hpi. (**B**) 12 hpi. Ten larvae were injected with CGMH-SL131 (1 × 10^4^ CFU) as the untreated group and later treated with 4e (6.25 mg/kg and 3.125 mg/kg) and TIG (tigecycline, 2 mg/kg) at specific post-infection timepoint. The percentage of larval survival rate was then monitored for 7 days. (**C**) Weight loss data in the mouse model, ns = not significant (*P* > 0.05) (**D**) Bacterial burden in the mouse kidney. Five mice were intraperitoneally injected with CGMH-SL131 (10^8^ CFU) and were treated with 4e (2.5 mg/kg and 1.25 mg/kg) at 1 h, 24 h, and 48 h. The kidney was harvested at 72 h to check for bacterial burden. All experiments were done in three biological and three technical replicates. ****P < 0.0001.

To assess the efficacy of 4e in rescuing *S. lugdunensis* infection *in vivo*, we first evaluated the toxicity of 4e in larvae. A 10 µL working stock solution at 1× MIC (3.125 mg/kg) and 2× MIC (6.25 mg/kg) was injected into the larvae, which were monitored for 7 days. Larvae treated with 4e alone demonstrated a 100% survival rate for five consecutive days and a 70% survival rate by day 7, comparable to the tigecycline-treated group (2 mg/kg) (Fig. S4A). By contrast, exposure to a high bacterial load (1 × 10^6^ CFU) of CGMH-SL131 was lethal, with only 40% survival observed on the first day (Fig. S4A). These data suggest 4e to be less toxic *in vivo*. In addition, 1 hpi (hour post-infection) showed that 6.25 mg/kg and 3.125 mg/kg of 4e rescued the larvae from CGMH-SL131 infection efficiently compared to the larvae group injected with 1 × 10^4^ CFU of CGMH-SL131 (an optimized CFU/mL that did not exhibit high larvae lethality) (Fig. 6A). We also investigated whether prolonged exposure to infection could influence the efficacy of 4e. To explore this, we treated the larvae with 4e at 12 hpi. The results revealed a 100% survival rate in the 6.25 mg/kg 4e treatment group, and a stable 80% survival rate from the third to the seventh day in the 3.125 mg/kg 4e treatment group (Fig. 6B). By contrast, the group injected with bacteria only showed a survival rate of 33.3%. These findings suggest that 4e can rescue larvae from lethal CGMH-SL131 infections. Therefore, 4e demonstrated efficient antibacterial activity in both *in vitro* and *in vivo* models.

### 4e partially decreased CGMH-SL131 kidney burden in the mouse peritonitis model

To further evaluate the *in vivo* efficacy of 4e against CGMH-SL131, C57BL/6 mice were intraperitoneally injected with 1 × 10^8^ CFU (lethal dose for larvae) and were administered with varying concentrations of 4e (2.5 mg/kg and 1.25 mg/kg) at 1 hpi, 24 hpi, and 48 hpi. The liver, kidneys, and heart were then aseptically harvested to assess bacterial burden at 72 hpi. Interestingly, unlike in the larvae infection model, CGMH-SL131 was prepared in a dose-dependent manner (10^6^ CFU and 10^7^ CFU) but failed to cause any mortality within 7 days in the mouse model (data not shown). Therefore, 1 × 10^8^ CFU of CGMH-SL131 was used for the mice peritonitis model (Fig. 6C and D). A stable weight was observed on 4e-treated mice up to 72 h, and weight loss was observed in the bacteria-injected group, only at 24 hpi (Fig. 6C). Mice eventually regained their weight in the next 48 h and 72 h. We next determined the bacterial colonization on visceral organs to determine whether 4e can potentially decrease the bacterial burden. The results showed that CGMH-SL131 colonized the murine kidney more efficiently than the liver and heart (Fig. S4B). Bacterial burden experiments revealed that mice without 4e treatment had kidney bacterial loads of 10^7^ CFU/kidney, whereas mice treated with 2.5 mg/kg and 1.25 mg/kg of 4e showed significantly reduced loads of 5.3 × 10^4^ and 1.3 × 10^5^ CFU/kidney, respectively (Fig. 6D). These results demonstrate that 4e effectively reduces CGMH-SL131 colonization in mouse kidneys.

### 4e significantly altered the metabolic profile of CGMH-SL131

To elucidate the potential mechanism of action, we mainly utilized untargeted metabolomics using the BEH amide and C18 columns. In these strategies, an investigation among hydrophilic and hydrophobic metabolites was conducted to investigate the mechanism of how 4e potentially inhibits cell growth and biofilm formation. Here, planktonic cells were allowed to grow to mid-exponential phase and were treated with 1/2 MIC (31.25 µg/mL), 2× MBIC_80_ (6.25 µg/mL), and DMSO (untreated control) for 1 h. Bacterial pellets and supernatant were then harvested and processed for UHPLC-ESI-QToF-MS/MS-based untargeted metabolomics analysis. Metabolite profiles of 4e-treated cells were then compared with the DMSO-treated group using a cut-off point fold-change of ≥1.5 (log2 1.5 = 0.58 in the y-axis). To identify the formulas of the metabolites, we used the Human Metabolome Database (HMDB) and the MiMeDB database. Tables S3 and S4 present the observed *m/z*, theoretical *m/z*, retention time, tentatively identified compound names and formulas, and mass errors (in ppm).

**Fig 7:**
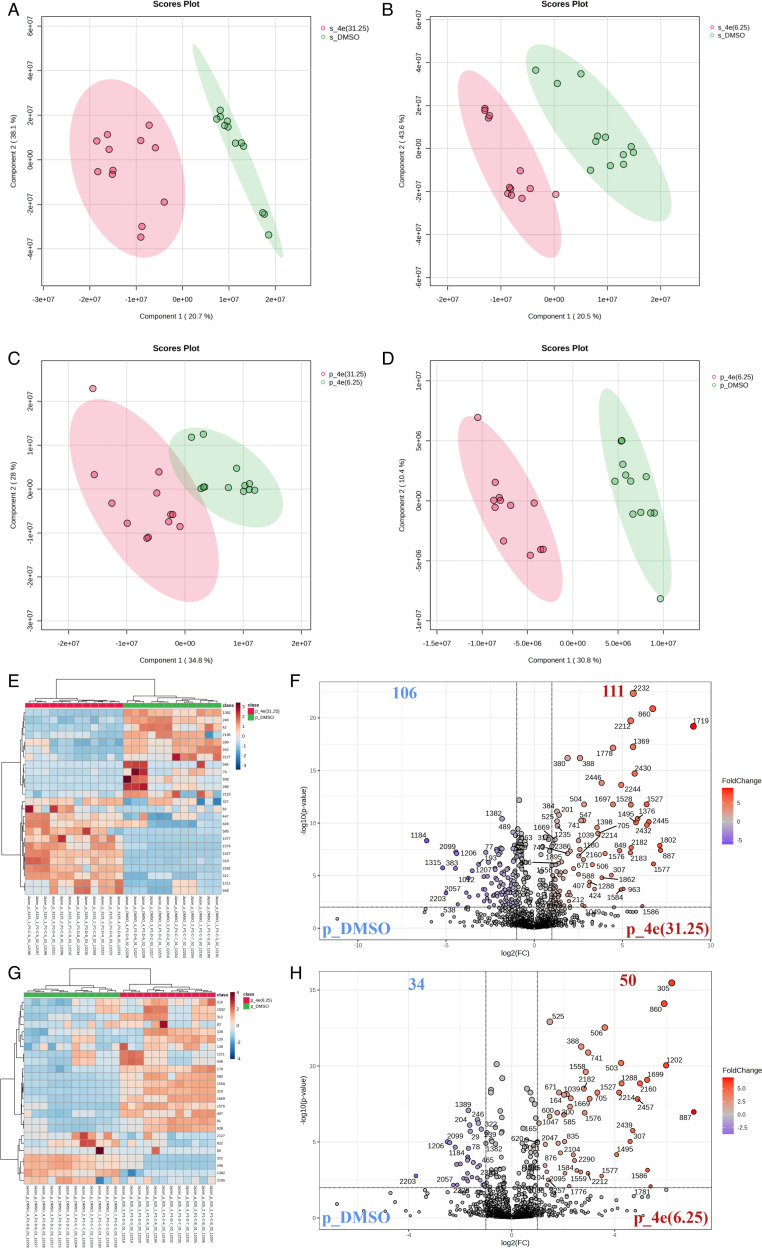
Untargeted metabolomics studies (amide column). (**A, B**) PLS-DA of the features obtained from the supernatant of 4e-treated groups (31.25 (**A**) and 6.25 (**B**) μg/mL) when compared to the DMSO-untreated group. (**C, D**) PLS-DA of the features obtained from the pellet of 4e-treated groups (31.25 (**C**) and 6.25 (**D**) μg/mL) when compared to the DMSO-untreated group. (**E**) Heat map of the top 25 features obtained from pellets with the highest scores from the PLS-DA and volcano plot (**F**) showing the number of differentially expressed features between 4e-treated groups (31.25 μg/mL) when compared to the DMSO-untreated group. (**G**) Heat map of the top 25 features obtained from pellets with the highest scores from the PLS-DA and volcano plot (**H**) showing the number of differentially expressed features between the 4e-treated groups (6.25 μg/mL) when compared to the DMSO-untreated group. Cut-off point of log2 fold-change ≤−1.5, log2 fold-change ≥1.5; and false detection rate (FDR < 0.05) was used in this experiment.

The results for the amide column revealed that 4e induced a remarkable metabolite alteration in all 4e-treated groups relative to the DMSO-treated group for both supernatant and pellet. PLS-DA revealed a separation of two distinct metabolite clusters between the 4e-treated and DMSO-treated groups (Fig. 7A through D). Hence, the observed metabolic alterations were highly driven by 4e treatment. In addition, an elevated amount of 4e (13.44-fold) was observed in the collected supernatant treated with 1/2 MIC, which confirmed the direct exposure of bacterial cells to 4e (Table S3A). However, the number of significant metabolite alterations in the supernatant was less than in the cell pellets (Table S3).

Importantly, 4e was detected in the collected pellet as evidenced by its significant increase in fold change (4.01) in the 1/2 MIC-treated group and a fold change of 2.83 in the 2× MBIC_80_-treated group (Table S3B). The data suggest that 4e from the supernatant can penetrate the cell, potentially affecting intracellular metabolic functions. For this reason, we focused our metabolite investigation mainly on the cell pellet. Heat maps and volcano plots in the cell pellet revealed significant differences in the metabolite profiles between the 4e-treated and DMSO-treated groups (Fig. 7E through H).

Volcano plots revealed that planktonic cells treated with 1/2 MIC of 4e generated more differentially expressed metabolites in the cell supernatant and pellet compared to the ones treated with 2× MBIC_80_ (Fig. S5B and D; Fig. 7E through H). A total of 111 metabolites were upregulated and 106 metabolites were downregulated under 1/2 MIC treatment (Fig. 7F), and 50 metabolites were upregulated and 34 were downregulated under 2× MBIC_80_ (Fig. 7H). In contrast to the supernatant, a total of 13 metabolites were upregulated and 38 metabolites were downregulated under 1/2 MIC treatment, and 11 metabolites were upregulated and 19 were downregulated under 2× MBIC_80_ concentration (Fig. S5B and D). Moreover, metabolite alterations were observed to be dose-dependent.

The same significant separation of two distinct clusters was observed using the C18 column based on their PLS-DA for both supernatant and pellet (Fig. S6A through D). However, less hydrophobic metabolites were observed to be differentially expressed in the C18 as compared to the hydrophilic metabolites in the amide platform. C18 in the supernatant shared similar results with the amide platform in the detection of 4e in high amounts (fold change of 8.98 under 1/2 MIC and 8.43 under 2× MBIC_80_) (Table S4).

By contrast, the cell pellet data were noted to have an upregulation of 60 metabolites and a downregulation of 74 metabolites under 1/2 MIC treatment. Once treated with 2× MBIC_80_, a total of 43 metabolites were upregulated and 13 metabolites were downregulated (Fig. S7A through D). In addition, we performed a two-sample t-test for the obtained metabolites with an FDR-adjusted *P*-value cut-off of 0.05 and a fold change of 2 to narrow down the potential lead metabolites of interest. Hence, 60 metabolites from the supernatant group and 198 from the pellet in the amide platform, as well as 32 from the supernatant group and 100 from the pellet in the C18, were removed due to low signal-to-noise ratios, poor peak shape, or the suspicion that they originated from the culture media.

**Fig 8:**
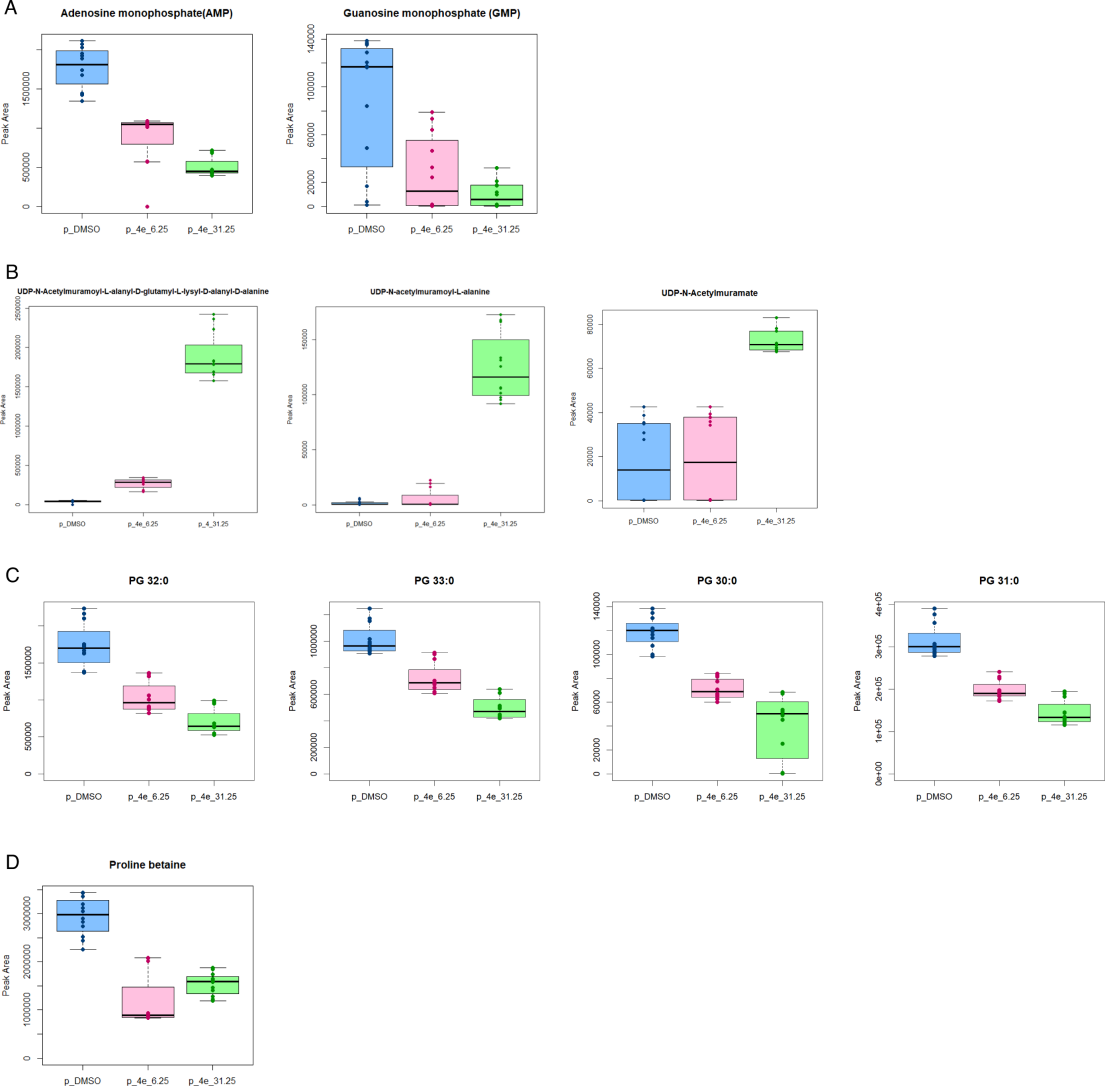
Box plots of metabolites of interest. (**A**) Downregulation of metabolites involved in the *de novo* purine biosynthesis was treated with both 1/2 MIC and 2× MBIC_80_ of 4e for 1 h. (**B**) Upregulation of metabolites involved in the cell wall biosynthetic process under 1/2 MIC 4e treatment for 1 h. (**C**) Downregulation of phosphatidylglycerols in the C18 column treated with both 1/2 MIC and 2× MBIC_80_ of 4e for 1 h. (**D**) Downregulation of proline betaine at 2× MBIC_80_ of 4e for 1 h. Cut-off point of log2 fold-change ≤−1.5, log2 fold-change ≥1.5; and false detection rate (FDR < 0.05) was used in this experiment.

Interestingly, the amide data revealed a significant downregulation in adenosine monophosphate (AMP) and guanosine monophosphate (GMP) under 1/2 MIC treatment (Fig. 8A). These results suggest that 4e may disrupt bacterial physiology that utilizes AMP and GMP in the biosynthetic process. Hence, 4e most likely interfered with the *de novo* purine pathway necessary for nucleotide synthesis and balance. In addition, a significant upregulation of three metabolites in the cell wall biosynthetic process was observed under 1/2 MIC treatment, which potentially predicted 4e’s ability to induce cell wall stress that may eventually affect peptidoglycan synthesis. Specifically, UDP-N-acetylmuramate, UDP-N-acetylmuramoyl-L-alanine, and UDP-N-acetylmuramoyl-L-alanyl-D-glutamyl-L-lysyl-D-alanyl-D-alanine were all detected to accumulate in the cell pellet (Fig. 8B). This disruption will potentially lead to a compromise of the cell wall synthesis and integrity, ultimately affecting the overall stability and function of the bacterial cell wall.

To further support the cell membrane targeting action, the C18 platform also revealed a downregulation of phosphatidylglycerols (PG), a class of phospholipids that are important components of bacterial cellular membranes. PG 30:0, PG 31:0, PG 32:0, and PG 33:0 were all observed to be downregulated (Fig. 8C). Moreover, in response to 1/2 MIC 4e treatment, the upregulation of arginine, argininosuccinic acid, and citrulline was observed, and these metabolites are crucial components of the urea (ornithine) metabolic pathway (Fig. S8A). In addition, N6, N6-dimethyladenosine (m6A), which plays a role in maintaining RNA stability and gene expression regulation, was also observed to be downregulated in both 1/2 MIC and 2× MBIC_80_ of 4e, respectively (Fig. S8B). Lastly, we observed a dose-specific downregulation of proline betaine, a known osmoprotectant, at 2× MBIC_80_ (Fig. 8B).

To validate the findings from metabolite profiling, we analyzed the RNA expression of genes involved in the *de novo* purine pathway and cell wall biosynthesis. Specifically, we examined the expression levels of enzymes encoded by *purA*, *purB*, *guaA*, and *guaB*, which play key roles in *de novo* purine biosynthesis and are responsible for producing purine nucleotides such as AMP and GMP, following 4e treatment. Our data showed a marked downregulation of *purA, guaA*, and *guaB* when cells were treated with 1/2 MIC of 4e (Fig. S9A). These results potentially support 4e’s role as a *de novo* purine biosynthesis inhibitor. In addition, *murA*, *murB*, *murC*, and *murD* genes catalyze the first steps in peptidoglycan synthesis, and these genes were also observed to be upregulated under 1/2 MIC 4e treatment (Fig. S9B).

Moreover, previous studies have shown that cell wall homeostasis and stress response upon β-lactam antibiotic exposure are under the control of a transmembrane protein, penicillin-binding protein and serine/threonine-associated (PASTA) kinase (22). Hence, we speculated that 4e may also bind to the PASTA kinase intracellular domain, affecting downstream signal transduction and thus disrupting cell wall homeostasis. Our preliminary *in silico* screening revealed a potential binding affinity of 4e in the PASTA kinase active site (Fig. S10). An enhanced oxacillin susceptibility was also observed when oxacillin in combination with a low concentration of 4e (12.5 µg/mL) was used to treat CGMH-SL131 planktonic cells for 24 h (Fig. S11).

## DISCUSSION

There is a growing demand for alternative therapies to address new emerging pathogens, with synthetic structure-based drug design being a key solution due to its scalability and diversity. This approach enables precise control over molecular structure and functional groups, allowing for the tailoring of drugs to enhance their efficacy (23). In this work, we highlighted the antibacterial and antibiofilm activities of our synthetic indolenine derivative 4e against a bacteremia isolate with high oxacillin resistance, CGMH-SL131. Importantly, we used untargeted metabolomic assays and RT-qPCR to identify potential targets of 4e in *S. lugdunensis* and uncover its potential antibacterial mechanisms of action.

Our study showed that compound 4e exhibited bacteriostatic activity against CGMH-SL131. However, even at 1× MIC, the required concentration of 4e was approximately 10 times higher than that of the antibiotic controls tested at 8× MIC. This indicates that 4e is less potent than tigecycline and daptomycin. These findings suggest that while 4e demonstrates antibacterial activity, its potency as a lead antibacterial agent is still relatively weak. Nonetheless, 4e structure modification can be done in the future to enhance antibacterial activity. The cell membrane and cell wall of bacteria are vital to their survival and pathogenesis. Thus, targeting these structural components remains one of the most widely employed strategies in drug development (24). By using live/dead fluorescence microscopy and SEM, we found that 4e could affect the cell membrane integrity and the maintenance of cell shape. At 2× MIC, approximately 26.7% of the bacterial population showed weakened membrane integrity, as indicated by PI fluorescence detection. In addition, the downregulation of PG 30:0, PG 31:0, PG 32:0, and PG 33:0, which are responsible for cell membrane structure and function, makes the cell more vulnerable to environmental stress and membrane-disrupting agents. Reports have shown that antimicrobial peptides can affect the physical properties of the cellular membrane through induction of membrane physical curvature, lipid clustering, prompting packing defects resulting in complete or partial loss of the permeability barrier, and by directly targeting components of a membrane, such as lipids (25). We also noticed a concerted upregulation of arginine, argininosuccinic acid, and citrulline under 4e treatment. Argininosuccinate is formed in the urea cycle by the reaction between citrulline and aspartate, and it is then converted into arginine and fumarate by the enzyme argininosuccinate lyase (26). Arginine is a precursor for the production of polyamines, such as putrescine, spermidine, and spermine. These polyamines help stabilize cell membranes, protect against oxidative stress, and support cell growth and division under stressful conditions (27). We hypothesized that the upregulation of these metabolites is part of the broader effort of the cell to maintain protein integrity, prevent oxidative damage, and repair membranes, especially when bacterial growth is threatened by 4e treatment. Thus, we found that 4e may alter the physical properties of the cell membrane, leading to its bacteriostatic effect.

Moreover, UDP-N-acetylmuramate, UDP-N-acetylmuramoyl-L-alanine, and UDP-N-acetylmuramoyl-L-alanyl-D-glutamyl-L-lysyl-D-alany are all intermediates in the biosynthesis of peptidoglycan, which is a key component of the bacterial cell wall (28). These molecules are involved in the sequential steps of building the peptidoglycan precursor, which is later incorporated into the cell wall structure. Our metabolomics data revealed a significant accumulation of these metabolites in the cell pellet. In addition, *murA*, *murB*, *murC*, and *murD* genes were also upregulated when treated with 4e. These findings indicate an adaptive response in which bacteria may compensate for stress by upregulating genes involved in peptidoglycan biosynthesis, thereby reinforcing cell wall assembly. Collectively, the results suggest that compound 4e disrupts bacterial cell wall integrity, potentially compromising structural stability and leading to increased cell membrane permeability.

Bacterial eukaryotic-like serine/threonine kinases (eSTKs) are structurally similar to eukaryotic protein kinases, containing the classic bi-lobed kinase domain and highly conserved Hanks kinase motifs (29). A subset of bacterial eSTKs contains different numbers of PASTA domains that have been shown to bind and detect cell wall fragments. PASTA kinases are found almost exclusively in single copy in gram-positive bacteria, such as *S. aureus*, *Listeria monocytogenes*, and even *Mycobacterium tuberculosis*, in which only 1 of 11 eSTKs is a PASTA-containing eSTK. PASTA kinases modulate several bacterial cell functions, including growth, cell wall remodeling, β-lactam resistance, central metabolism, biofilm formation, and virulence (30, 31). Recently, Schaenzer et al. demonstrated that a pyrazolopyridazine derivative (GW779439X) sensitizes MRSA to various β-lactams through the inhibition of PASTA kinase Stk1. Compound GW779439X potentiates β-lactam activity against multiple MRSA and MSSA isolates, including the resensitization of a ceftaroline-resistant isolate to ceftaroline (32). Furthermore, phosphoproteomic analysis provided evidence of the PASTA kinase (PrkA)-dependent control of peptidoglycan synthesis via ReoM in *L. monocytogenes* (33). It was shown that ReoM is the direct target of PrkA that influences peptidoglycan synthesis and repair under cell wall stress, and this phenomenon facilitated cytosolic survival and virulence *in vivo*. Hence, screening for PASTA kinase inhibitors is an attractive alternative treatment for bacterial infections. We performed *in silico* screening and demonstrated the high binding affinity of 4e to the PASTA kinase active site (−9.8 kcal/mol) of *S. lugdunensis* (data not shown). However, we did not observe complete oxacillin potentiation in CGMH-SL131, but somehow, we noticed an enhanced susceptibility. Therefore, further investigation is needed to determine whether 4e directly binds to the PASTA kinase of *S. lugdunensis* to inhibit its activity.

In most bacteria, nucleotides are synthesized *de novo* and play critical roles in various cellular functions, such as DNA replication, energy storage, and cellular signaling (34). These pathways are vital for maintaining the overall metabolic balance of the cell. Hence, purine and pyrimidine nucleotide biosynthesis pathways are linked to the virulence of various opportunistic and primary pathogens (35). Disruptions in these pathways can impact a bacterium’s ability to replicate, respond to environmental stress, and regulate key cellular processes, thereby influencing its pathogenicity and ability to cause disease (35). A previous work demonstrated that purine biosynthesis is required for intracellular growth of *S. aureus* and hypervirulence phenotype (36). Moreover, microarray analysis further revealed that purine biosynthesis impacts the expression of 400 genes involved in a broad spectrum of functions, including virulence. The purine biosynthesis mutant strains in *purA* and *purH* exhibited significant attenuation of virulence in a murine abscess model (37).

For translational context, a recent work demonstrated that 6-thioguanine (6-TG), a purine analog secreted by *Staphylococcus chromogens* ATCC 43764, suppresses *S. aureus* growth by inhibiting *de novo* purine biosynthesis (38). When 6-TG was used prophylactically, it exhibited reduced necrotic skin lesions in mice infected with *S. aureus* USA300. This result was supported by their observation that 6-TG reduces alpha-toxin production. In addition, 6-TG downregulates the expression of genes coding for purine biosynthesis, the accessory gene regulator (*agr*), and ribosomal proteins in *S. aureus*, explaining its anti-virulence activities. Our 4e small molecule may act similarly to 6-TG as it exhibited bacteriostatic activities and disruption in purine biosynthesis. Although we did not perform experiments to show direct evidence of toxin production inhibition and a murine skin infection model, the downregulation of m6A denotes a possible reduced gene expression of virulence factors like the production of bacterial toxins. The m6A is a methylation modification on adenosine residues in RNA, and methylation plays an essential role in regulating mRNA stability, translation efficiency, and gene expression (39). We hypothesized that 4e affects RNA modification pathways, potentially inhibiting the activity of enzymes like methyltransferases. This could result in altered gene expression or reduced stability of certain RNA molecules. Moreover, the downregulation of m6A might be an indirect consequence of changes in methionine cycle metabolism or SAM (S-adenosylmethionine) metabolism, which are responsible for providing the methyl groups required for RNA modifications (40, 41). Our data also revealed methionine downregulation in cells treated with 1/2 MIC of 4e.

It is also worth knowing that there are extensive studies on the regulation of bacterial biofilms via purine metabolism (42, 43). A recent transcriptomic analysis, validated with transposon mutants, showed that genes associated with the *de novo* purine biosynthesis pathway were the only consistently upregulated genes in biofilms of both *S. aureus* and *E. faecalis* when grown in a TSB-based medium (42). *S. aureus* USA300 strains with transposon (Nebraska Transposon Mutant Library; NTML) insertion in *purA*, *purD*, *purH*, *purM*, *purN*, and *purQ* genes from the purine biosynthesis pathway showed weak biofilm formation, highlighting purine biosynthesis as an interesting new antibiofilm target. Hence, the potential action of 4e in disrupting *de novo* purine biosynthesis demonstrated in our work suggests a potential mechanism of action towards its antibiofilm potential. Without sufficient adenosine triphosphate (ATP) and guanosine triphosphate (GTP) derived from AMP and GMP, bacterial growth and proliferation will be impaired. As a result, fewer cells can initiate biofilm formation.

Moreover, cyclic-di-GMP (c-di-GMP) is a second messenger used by many bacteria to regulate processes like biofilm formation, motility, and virulence (44). It was known that c-di-GMP is synthesized from GTP, which can be derived from GMP (45). Hence, we hypothesized that 4e inhibits bacterial adhesion to surfaces and stimulates biofilm dispersal by disrupting the c-di-GMP signaling pathway, potentially through the inhibition of purine metabolism, particularly by downregulating GMP synthesis and indirectly reducing GTP availability, thereby limiting c-di-GMP synthesis. Specifically, 4e may target the *guaA* gene, leading to reduced GMP and indirectly lowering GTP, which in turn reduces c-di-GMP synthesis, promoting biofilm dispersal (46). Moreover, reports have shown that proline betaine contributes to biofilm maturation in *S. aureus* by enabling the bacteria to manage osmotic stress, enhance their survival, and form resilient biofilms, which complicates treatment efforts for infections caused by this pathogen (47). Therefore, 4e may potentially target proline betaine, which could weaken the biofilm structure and integrity, making it prone to biofilm dispersal. Furthermore, the biofilm dispersal activity of compound 4e is comparable to that of proteinase K, a well-known serine protease capable of degrading the biofilm matrix (44). This suggests that 4e may serve as an effective biofilm matrix-disrupting agent. However, to gain deeper insight into its mechanism of action, a multi-omics analysis comparing treated and untreated biofilm samples is essential in the future.

Importantly, the transcriptional profile of *S. aureus* revealed a strong regulatory impact of PknB (PASTA kinase) on the expression of genes encoding proteins that are involved in purine and pyrimidine biosynthesis and cell wall metabolism (48). Thus, the downregulation of AMP and GMP metabolites, as well as the reduced gene expression of *purA*, demonstrated in our work, could be a putative mechanism of action toward inhibiting and disrupting biofilms that may be under the control of the PASTA kinase signaling pathway. Our current work opens more future research opportunities to further understand how PASTA kinase is linked to purine biosynthesis and cell wall maintenance, which may control *S. lugdunensis* pathogenesis and antibiotic resistance. To date, no extensive studies have used *S. lugdunensis* in this context. Thus, this limits our *S. ludgunensis*-based literature review and result comparison. However, since *S. lugdunesis* is highly associated with infective endocarditis (49, 50), it is worth investigating the association of purine metabolism or PASTA kinase pathways in endocarditis models in the future.

In our *in vivo* investigation, we noticed that a high concentration of CGMH-SL131 (1 × 10^8^) was not highly virulent to the murine model compared to the larval model since we did not observe mortality cases among the five tested mice. A report has shown that the striking difference between *S. lugdunensis* and *S. aureus* is the absence of any obvious immune evasion molecules (51). The lack of these molecules and other virulence factors found in *S. aureus* in *S. lugdunensis* explains its low infectivity *in vivo*. This observation was comparable with the previous work, which investigated the function of heme and non-heme iron acquisition mechanisms in *S. lugdunensis* HKU09-01 *in vivo*, using a murine infection model (52). Furthermore, Flannagan et al. observed that *S. lugdunensis* HKU09-01 displayed organ-specific differences in bacterial proliferation and indicated that the kidney is the most suitable organ to support significant bacterial growth because of the readily available iron in the organ for HKU09-01 nutritional source. In our study, all five mice infected with CGMH-SL131 showed higher bacterial burden in the kidney compared to the liver, spleen, and heart. Notably, three mice in the CGMH-SL131-infected group consistently showed a high bacterial burden, while three mice treated with 4e exhibited a 3-log10 reduction in bacterial load in their kidneys. The results of both the mice and larvae infection models provide preliminary proof of concept, which can support 4e’s *in vivo* efficacy.

Small compounds were previously studied in a dose-dependent manner in murine models with a starting concentration of 10 mg/kg (53, 54). We attempted to adopt this type of approach, but we observed solubility issues of our 4e stock (10 mg/mL) dissolved in DMSO when mixed with aqueous solutions (1× PBS) at ≥5 mg/kg. In addition, *in vitro* cytotoxicity toward human-derived cell lines showed high cytotoxicity at 4× MIC (250 µg/mL). These results made us ponder that solubility issues in an aqueous solution and cytotoxicity at high concentrations limit us to investigate 4e in its full therapeutic potential. Nonetheless, sub-MIC concentration seems more feasible for future investigations since no cytotoxicity and solubility issues were observed.

In conclusion, this study demonstrated the potential application of indolenine-substituted pyrazole, 4e, as a potent antibiofilm agent targeting *S. lugdunensis* biofilm. In addition, 4e’s capability of compromising cell wall and bacterial membrane integrity and disrupting *de novo* purine biosynthesis needs further validation. Further investigations, including more targeted studies on its interactions with PASTA kinase, purine metabolism pathways, RNA modification pathways, and murine skin infection models, are necessary to optimize and understand its therapeutic potential. Moreover, the synthesis of second-generation small molecules based on the 4e chemical backbone will be prioritized to identify new indolenine-based compounds with enhanced antibacterial activity, improved solubility, and no cytotoxicity. In future studies, 4e and its derivatives may also be combined with bactericidal agents to accelerate bacterial killing in both planktonic and biofilm states. Lastly, 4e could be formulated for topical application on implantable medical devices such as wound dressings, surgical sutures, or catheter insertion sites to potentially inhibit bacterial colonization and biofilm formation, thereby reducing the risk of device-associated infections.

## MATERIALS AND METHODS

### Bacterial strains and cell lines

Oxacillin-resistant bacteremia isolate *S. lugdunensis* CGMH-SL131 and other clinical strains coded with CGMH (CGMH-SL20, CGMH-SL134, CGMH-SL104, CGMH-SL138, CGMH-SL99, CGMH-SL61, CGMH-SL144, CGMH-SL49, CGMH-SL33, CGMH-SL19, CGMH-SL36, CGMH-SL52, CGMH-SL118, CGMH-SL139, CGMH-SL190, CGMH-SL864, CGMH-SL868, CGMH-SL872, CGMH-SL879, CGMH-SL880, CGMH-SL11, CGMH-SH53, CGMH-SA1205, and CGMH-SA2) were obtained from the Department of Laboratory Medicine, Linkou Chang Gung Memorial Hospital in Taoyuan, Taiwan. *S. aureus* strains 29213 and 25923 were obtained from the American Type Culture Collection (ATCC) (Manassas, VA, USA). *S. aureus* USA300 LAC, a community-acquired USA300 MRSA strain, was a gift from Dr. John-Demian Sauer (University of Wisconsin-Madison). *S. aureus* NTUH-SA1, *S. saprophyticus* TVGH-94-2, *S. epidermidis* NTUH-01, *S. agalactiae* (Group B) TVGH-58-2, *E. faecalis* TVGH-24-2, *E. raffinosus* TVGH-56-2, vancomycin-resistant *E. faecium* NTUH-EF4, carbapenem-resistant *P. aeruginosa* NTUH-PA06, carbapenem-resistant *K. pneumoniae* NTUH-KP4, and carbapenem-resistant *E. coli* NTUH-EC13 were all obtained from National Taiwan University Hospital. Carbapenem-resistant *A. baumannii* NCKH-AB9291 was obtained from National Cheng Kung University Hospital. Bacterial strains were prepared in cationic-adjusted Mueller-Hinton broth (CAMHB) or Trypticase Soy broth/agar (TSB/TSA) and cultured at 37°C.

Immortalized keratinocyte cell line, HaCaT, colon adenocarcinoma cell line, Caco-2, and embryonic kidney cells HEK293 were maintained in DMEM medium (Gibco-BRL, USA) supplemented with 20% heat-inactivated fetal bovine serum (FBS) (Gibco-BRL). The lung cancer cell line, H1975 CRL-5908, was maintained in RPMI medium (Gibco-BRL) supplemented with 10% heat-inactivated FBS. Cells were cultured at 37°C in a 5% CO_2_ atmosphere.

### 4e compound, antibiotics, and proteinase K

4e was synthesized by our group as previously described (21). The starting materials for the synthesis of 4e were purchased from Sigma-Aldrich Co. (Missouri, USA) and Merck KGaA (Darmstadt, Germany). The crude product was purified by column chromatography over silica gel using varying ratios of hexane and ethyl acetate (7:3-1:1 vol/vol) as the mobile phase. The purity of the compound was checked by high-performance liquid chromatography (HPLC) analysis. A 4e-stock concentration (10 mg/mL) was dissolved in DMSO. Lyophilized powder of tigecycline (Wyeth Lederle S.r.l., Italy) and daptomycin (WonWon Biotechnology Co., Ltd, Taiwan) were dissolved in DMSO to make the stock solution (10 mg/mL). Proteinase K (WonWon Biotechnology Co., Ltd, Taiwan) was dissolved in sterile MQ water to make a 1 mg/mL stock solution. According to EUCAST guidelines, the MIC breakpoint for tigecycline against *Staphylococci* is defined as susceptible (S) at ≤0.5 mg/L and resistant (R) at >0.5 mg/L. By contrast, CLSI guidelines define susceptibility and resistance for daptomycin as S: ≤1 mg/L and R: >1 mg/L (55, 56).

### Growth inhibition percentage and colony-forming unit count

The MIC of 4e for each bacterial strain was determined by the broth microdilution method as previously described with modifications (32, 57). Briefly, 24 h bacterial cultures (OD_600_ = 1.0) in CAMHB were back diluted (1:100) in fresh CAMHB (equivalent to 10^7^ CFU/mL) in a 96-well plate. The bacterial strains were then exposed to different concentrations of 4e (ranging from 7.81 to 125 µg/mL) in triplicate for 24 h at 37°C with constant agitation at 250 rpm. Tigecycline (6.25 µg/mL) and daptomycin (6.25 µg/mL) were used as positive controls, and 1% DMSO was used as a negative control. The bacterial density was measured (OD_600_) using the BioTek Synergy HTX Multimode Reader (Agilent Technologies, USA) at a 24 h endpoint. Growth inhibition percentage was computed using the equation: % growth inhibition = Abs_600_(negative control) − Abs_600_(treatment)/Abs_600_(negative control) × 100. The MIC of each agent was defined as the lowest concentration with at least ≥90% growth inhibition and with no visual broth turbidity (bacterial growth). CFUs were enumerated every 6 hours for 24 h to determine if the compound has bacteriostatic or bactericidal activity. One hundred microliters of the sample were taken, followed by 10-fold dilutions in 1× PBS, and 20 µL from various dilutions was spread on TSA plates and incubated at 37°C until CFUs were evident. All assays were performed in biological and technical triplicate.

### Multi-passage resistance assay

To assess whether CGMH-SL131 develops resistance to 4e, a serial passaging method was employed, with MIC determination (58). Briefly, overnight culture of CGMH-SL131 was diluted 1:200 in CAMHB medium, and 4e was added to achieve twofold serial dilution from 2× MIC until sub-MICs (1/2, 1/4, and 1/8 MIC). Bacterial growth in CAMHB medium without 4e served as the control. Cultures were incubated at 37°C with agitation at 140 rpm for 18–24 h. After each passage, the MIC was re-determined. If an increase in MIC was observed, a new 1/2 MIC concentration of 4e was established for the next passage. This process was repeated for 20 passages to assess changes in MIC values that denote potential resistance over repeated exposure. Rifampicin treatment was employed as a positive control.

### Cell membrane disruption assay

A 24 h broth culture of CGMH-SL131 was adjusted to OD_600_ = 0.5 and was exposed to 1% DMSO (negative control), 0.5% SDS (positive control), and 4e (1× MIC and 2× MIC) for 24 h at 37°C. The treated cells were harvested by centrifugation (13,000 rpm for 3 min) and resuspended in 200 µL PBS. The cell suspension was added with a dye mixture containing propidium iodide (10  µg/mL) and Syto9 (3 µM) dyes and incubated for 15 min in the dark. The dyed cells were harvested by centrifugation (13,000 rpm for 3 min) and resuspended in 200 µL PBS. The 100 µL was then transferred to a 24-well plate and allowed to dry, and cell viability was observed using a fluorescence microscope (IX83 Olympus Inverted Fluorescence Microscope, USA) equipped with 40× magnification (59). The experiment was done in three biological replicates with two technical replicates. In addition, a 96-well plate setup was adapted from previous studies, with modifications for fluorescence intensity detection of Syto9 (Ex 480 nm/Em 500 nm) and PI (Ex 535 nm/Em 617 nm) using a multimode microplate reader (TECAN Infinite 200/200) (60, 61). Relative fluorescent signals, obtained after background fluorescence subtraction, were used to calculate the percentage of live and dead cell populations as well as the live:dead ratio. The formula used was as follows:

% Live cells = (green fluorescence/[green fluorescence + red fluorescence]) ×100

% Dead cells = (red fluorescence/[red fluorescence + green fluorescence]) × 100 live:dead ratio = % live cells/% dead cells

The experiment was done in biological and technical triplicate.

### Membrane potential assay

CGMH-SL131 was grown to exponential phase and suspended to OD_600_ = 0.5 in 1× PBS with 30 µM 3,3′-diethyloxacarbocyanine iodide (DiOC2(3), Molecular Probes) and was incubated at 37°C for 15 min in the dark and subsequently treated with 2× MIC and 1× MIC of 4e for 60 min. The protonophore carbonyl cyanide m-chlorophenyl hydrazone (CCCP, Sigma Aldrich) at a concentration of 5 µM was used as a positive control, and 1% DMSO was used as a negative control. Fluorescence was measured at an optimal excitation wavelength of 485 nm and two emission wavelengths, 530 nm (green) and 630 nm (red), using a microplate reader (TECAN Infinite M200). Afterward, the 630 nm/530 nm ratio and the percentage of fluorescence intensity were calculated (62). The experiment was done in biological and technical triplicate.

### Scanning electron microscopy analysis

To determine cell morphology disruption, CGMH-SL131 was grown to exponential phase and suspended to OD_600_ = 0.5 in 1× PBS and was exposed to different concentrations of 4e (2× MIC, 1× MIC), 1% DMSO (negative control), and 0.5% SDS (positive control), for 24 h incubation at 37°C. After the treatment, cells were washed twice with 1× PBS and centrifuged (13,000 rpm for 3 min) to pellet the cells. The pelleted cells were fixed with 2.5% (vol/vol) glutaraldehyde for 12 h at 4°C. After which, the cells were subjected to alcohol dehydration with a gradient concentration of ethanol (30%, 50%, 70%, 90%, and 100%) at 10 minute intervals. Then, the dehydrated cells were resuspended in 50% ethanol, and an aliquot of 5 µL was transferred to an 8 mm cover glass and allowed to dry. The dried cells were soaked in 500 µL of 100% ethanol and subjected to critical point drying with liquid CO_2_. The cells were viewed using field emission scanning electron microscopy (SEM) (JEOL JSM-7600F, Japan). On the other hand, to assess biofilm inhibition, 24 hour bacterial cultures in TSB were diluted 1:100 in fresh TSB (resulting in 10⁷ CFU/mL). A 500 µL aliquot of the bacterial suspension was supplemented with the desired concentration of 4e (2× MIC, 1× MIC) and added to individual wells of a 24-well plate, each containing a cover glass slide (approximately 8 mm) placed at the center of each well. The plate was incubated at 37°C for 24 h to allow biofilm formation on the cover glass. The medium containing the planktonic cells was removed, and the wells were washed once by 1× PBS. It was then followed by 2.5% (vol/vol) glutaraldehyde fixation and alcohol dehydration, which were the same steps mentioned above. Then, the dehydrated cells were soaked in 500 µL of 100% ethanol and were subjected to critical point drying with liquid CO_2_. Biofilm inhibition was then observed using field emission SEM (JEOL JSM-7600F). These experiments were adapted from previous works with modifications in three biological and two technical replicates (63, 64).

### Cytotoxicity assay against human cell lines

The cytotoxicity of different concentrations of 4e (2× MIC, 1× MIC, 1/2 MIC, and 1/4 MIC) was evaluated using the 3-(4,5-Dimethylthiazol-2-yl)−2,5-diphenyltetrazolium bromide (MTT; Sigma-Aldrich, St Louis, MO, USA) assay according to our previous study (65). Seeding of cells was performed in a sterile 96-well plate at an optimized concentration of 3.5 × 10^5^ cells/mL in 100 µL volume and was incubated at 37°C with 5% CO_2_ for 2 days to generate mature growing cells. The medium was replaced by a new medium containing different concentrations of 4e (2× MIC, 1× MIC, and sub-MICs), 0.1% Triton X-100 (toxicity control), and 1% DMSO (non-toxic control), and incubated for another 24 h using the same parameters. The next day, the medium was removed, and 30 µL of MTT reagent (0.5 mg/mL) was added to each well, and the plates were incubated at 37°C for 5 h in the dark. Then, 200  µL of 100% (vol/vol) DMSO was added to each well, and the plate was incubated with shaking (150 rpm) at room temperature for 30 min to allow the colorimetric reaction. The OD was measured at 570 nm using the BioTek Synergy HTX Multimode Reader. The percentage of cell viability was calculated as follows: % Cell viability = Abs_595_(treated cells) − Abs_595_(blank)/Abs_595_(negative control) − Abs_595_(blank) × 100. The experiment was done in biological and technical triplicate.

### Biofilm formation, inhibition, and dispersal assay using crystal violet staining

The 96-well flat plate (Greiner; 655185, Sigma-Aldrich) method and 0.1% crystal violet (CV) staining were used to screen all the isolates for their ability to produce biofilms (21, 66). The overnight culture mixture was diluted 1:100 with fresh TSB, and 200 µL of this diluted culture mixture was inoculated into individual wells of 96-well flat-bottom plates in triplicate and incubated at 37°C for 24 h. After 24  h of incubation at 37°C without agitation, the broth containing the planktonic cells was removed, and wells were washed with 1× PBS. Wells containing washed biofilms were fixed with 100% (vol/vol) methanol for 20 min. Methanol was then removed, and the wells were subjected to air drying for 15 min. Then, 100 µL of 0.1% CV was added to each well to stain the biofilms and incubated at room temperature for 20 min. The wells were washed twice with 1× PBS to remove unbound CV and were air-dried for 15 min. The CV-stained biofilm was resuspended with 200 µL of 95% (vol/vol) ethanol, and OD_595_ absorbance was measured using spectrophotometry (BioTek Synergy HTX Multimode Reader). Untreated cells were considered as negative controls, and wells containing TSB only were considered as blanks. The cut-off value (ODc) was used to categorize the isolates as strong biofilm producers or not. Where ODc  =  average OD of the negative control + [3× standard deviation (SD) of the negative control]. Then, the following criteria were used to describe the biofilm-forming capacity: OD ≤ ODc, non-biofilm forming; ODc <OD ≤ 2 ×ODc, weak biofilm-forming; 2 × ODc < OD ≤4 × ODc, moderate biofilm-forming; 4 × ODc < OD, strong biofilm-forming. To determine the biofilm inhibition, we used the same steps mentioned above; however, the bacterial culture was incubated together with 4e and proteinase K (2 µg/mL). In brief, a 24-h-old CGMH-SL131 in TSB was back diluted (1:100) in fresh TSB (equivalent to 10^7^ CFU/mL), and 200 µL of bacterial suspension was supplemented with the desired amount of 4e (ranging from 1.56 to 6.25 µg/mL), and proteinase K was added into individual wells in a flat-bottomed 96-well plate. Biofilm dispersal assay followed the same steps, but first, biofilms were allowed to form for 24 h incubation at 37°C without agitation. Then, wells containing preformed biofilms were washed with 1× PBS and were exposed to 4e (2× MIC, 1× MIC, 2× MBIC_80_, 1× MBIC_80,_ and 1/2 MBIC_80_), daptomycin (8× MIC) and proteinase K (2 µg/mL) for another 24 h at 37°C without agitation. Fixation in methanol and staining with 0.1% CV proceeded, following the same procedure as the biofilm inhibition assay. The percentage of biofilm formation was calculated as follows: % Biofilm formation = Abs_595_(treated cells) − Abs_595_(blank)/Abs_595_(negative control) − Abs_595_(blank) × 100. The experiment was done in biological and technical triplicate.

### Killing activity against combined planktonic and biofilm bacterial cells following treatment

An overnight culture of CGMH-SL131 was diluted 1:100 into a TSB medium. Then, the biofilm was formed for 24 h. After this, planktonic cells were removed and replaced with aliquots of 4e (2× MIC, 1× MIC, 1× MBIC_80_, 1/2 MBIC_80_) and proteinase K (2 µg/mL) concentrations and incubated for 24 h. As a positive control, wells with no 4e or proteinase K were used, and daptomycin (8× MIC) was added as an antibiotic-treated group. The wells were sonicated for 20 seconds in an ultrasonic bath, followed by mechanical disruption of the biofilms using a pipette tip to scratch the well surface. The contents were then pipetted up and down to ensure complete detachment of the biofilms. Subsequently, 100 µL of the suspension was serially diluted 10-fold, and the diluted samples were plated on TSA and incubated at 37°C for 24 h to determine bacterial CFU counts. The experiment was done in three biological and technical triplicates.

### Larvae infection model

*Galleria mellonella* larvae infection test was carried out based on the previous report (67). The larvae weighing around 0.2 g were selected in this assay. Overnight cultures of CGMH-SL131 established in CAMHB were harvested by centrifugation, washed, and suspended in sterile 1× PBS to an OD_600_ of 1 in 1× PBS. A final bacterial concentration of 3 × 10^6^ CFU/mL was used in this infection model. Each set of 10 larvae received a 10 µL bacterial suspension injection into the second-to-last left proleg. 4e (6.25 mg/kg and 3.125 mg/kg) and tigecycline (2 mg/kg) were injected in two independent experiments at 1 and 12 hpi. In addition, control larvae received the same volume of sterilized 1× PBS. Larvae were kept at 37°C in the dark without feeding for 7 days, and the percentage of survival rates was recorded every 24 h until 7 days. The larval infection was performed in biological and technical triplicate.

### Mice peritonitis model

Female C57BL/6 mice (6–8 weeks of age) were purchased from the National Applied Research Laboratories (Taipei, Taiwan) and housed as groups under conditions of constant photoperiod (12 h light, 12 h dark) with *ad libitum* access to sterilized food and water in the Animal Biosafety Level 2 laboratory of the Animal Center, National Yang Ming Chiao Tung University. All experimental procedures with these mice were performed following the protocols approved by the Institutional Animal Care and Use Committee of the National Yang Ming Chiao Tung University (approval number 11302206). Briefly, overnight-grown CGMH-SL131 in CAMHB was harvested by centrifugation, washed, and suspended in sterile 1× PBS to an OD_600_ of 1.5. Mice were infected by intraperitoneal administration of CGMH-SL131 (0.2 mL of bacterial suspension equivalent to 1 ×  10^8^ organisms) through a 1 mL syringe with a 25-gauge needle. The same bacterial culture was plated onto TSA to confirm the number of organisms inoculated. Five mice were assigned to three treatment groups and one untreated group. Each mouse subsequently received treatment at 1, 24, and 48 hpi with two varying concentrations of 4e (2.5 mg/kg and 1.25 mg/kg). Mice that did not receive any 4e treatment were used as controls. Mice were sacrificed by carbon dioxide euthanasia at 72 h. Organs were aseptically harvested into 1.5 mL PBS with 0.1% (vol/vol) NP-40, homogenized, diluted, and plated onto TSA to enumerate bacterial burden. Recovered bacterial load from organs is presented as log10 CFU per organ.

### Sample processing for untargeted metabolomics

Sample preparation for untargeted metabolomics was prepared based on previous reports with modification (68). In brief, an overnight CGMH-SL131 was refreshed at 1:100 dilution with fresh CAMHB up to mid-log phase (OD_600_ = 0.5, approximately 5 h at 37°C, 200 rpm), and was treated with 4e (sub-MIC and 2× MBIC_80_) and DMSO for 1 h. After treatment, the supernatant and pellet were separated by centrifugation (8,000 rpm for 5 min) at 4^ο^C. To process the obtained supernatant, 100 µL was mixed with 400 µL of ice-cold methanol to quench proteins. Protein precipitation was carried out by centrifugation at 30,000 × *g* for 15 min at 4°C. The resulting supernatant was then filtered through a 0.22 µm regenerated cellulose membrane (RC-4, Sartorius, Göttingen, Germany). On the other hand, the bacterial pellets were washed twice with 400 µL of ice-cold PBS to remove residual supernatant. Each wash was followed by centrifugation at 3,552 × *g* for 5 min. To quench proteins, 100 µL of MS-grade water and 400 µL of ice-cold methanol were added to the washed bacterial pellets. Protein precipitation was achieved by centrifugation at 30,000 × *g* for 15 min at 4°C. Supernatant and pellet samples were stored at −20°C until LC-MS analysis.

### Instrumentation and data analysis

We adapted a previous work for the untargeted metabolomics workflow with modifications (69). In brief, we employed an Agilent 1290 ultra-high-performance liquid chromatography (UHPLC) system (Agilent Technologies, Waldbronn, Germany) coupled with a Bruker maXis ultra-high-resolution (UHR) time-of-flight (TOF) mass spectrometer (Bruker Daltonics, Bremen, Germany). For the separation of polar metabolites, a bridged ethylene hybrid (BEH) amide column (2.1 mm × 100 mm, 1.7 µm) was used with a gradient elution method. Mobile phase A consisted of 10 mmol/L ammonium acetate and 0.1% formic acid in deionized water, while mobile phase B contained 10 mmol/L ammonium acetate and 0.1% formic acid in 95% acetonitrile and 5% water. The gradient elution was performed at a flow rate of 0.4 mL/min under the following conditions: 0–0.5 min, 99% mobile phase B; 0.5–7 min, 99%–50% mobile phase B; 7–10 min, 50% mobile phase B, followed by a 2 minute re-equilibration using 99% mobile phase B. We utilized an Agilent Infinity Lab Poroshell HPH-C18 column (2.1 × 100 mm, 1.9 µm) and a gradient elution method to separate nonpolar metabolites and lipids. Mobile phase A consisted of deionized water containing 10 mmol/L ammonium acetate and 0.1% formic acid, while mobile phase B was a mixture of methanol and isopropanol (2:3 ratio) containing 10 mmol/L ammonium acetate and 0.1% formic acid. The gradient elution was conducted at a flow rate of 0.4 mL/min with the following conditions: 0-3 min, 0%–100% mobile phase B; 3–10 min, 100% mobile phase B, followed by a 2 minute re-equilibration using 0% mobile phase B. The injection volume was 10 µL, with the autosampler and column oven maintained at 4°C and 40°C, respectively. Electrospray ionization in positive ionization mode was used, with parameters set as follows: dry gas temperature, 200°C; dry gas flow rate, 8 L/min; nebulizer pressure, 2 bar; capillary voltage, 4,500 V; and endplate offset, 500 V. Mass spectra were recorded in the range of 50–1,500 m/z, and the TOF mass analyzer was calibrated with sodium formate over a 50–1,500 m/z range before analysis to ensure accuracy. Each sample was analyzed in biological and technical triplicate to ensure reliability and reproducibility. Furthermore, maXis UHR-TOF data were processed using two software tools: MS-DIAL version 4.92 (available at http://prime.psc.riken.jp/) and Bruker Compass DataAnalysis software version 4.1. Peak detection and alignment were performed with a minimum peak height threshold of 10,000, a retention time tolerance of 0.5 min, and an MS1 tolerance of 0.05 Da. All other parameters were set to their default recommended values. The data were normalized for statistical analysis using autoscaling, which involves mean-centering and scaling each variable by its standard deviation. Analytical techniques such as principal component analysis (PCA), partial least squares discriminant analysis (PLS-DA), heatmaps, and volcano plots were applied using MetaboAnalyst 6.0, a web-based metabolomics analysis platform accessible at https://www.metaboanalyst.ca/. The identification of known features was verified using a custom compound library constructed with commercial reference standards, containing details such as retention times, m/z values, and MS/MS fragment spectra. For annotating unknown features of interest, databases including the Human Metabolome Database, the Human Microbial Metabolome Database, MS-DIAL metabolomics MSP, and MS-DIAL LipidBlast were employed.

### RNA extraction and reverse transcription quantitative PCR

An overnight bacterial culture of CGMH-SL131 was refreshed at 1:100 dilution with fresh CAMHB up to mid-log phase, approximately 5 h at 37°C and 200 rpm (OD_600_ = 0.5), and was treated with 4e (1/2 MIC and 2× MBIC_80_) and DMSO for 1 h. Cells were separated by centrifugation (8,000 rpm for 5 min) at 4°C. Pellet cells were then subjected to cell lysis by adding 100 µL of lysis agent containing lysozyme (40 µg/mL) and lysostaphin (200 µg/mL) for 1 h. RNA extraction was then performed according to the manufacturer’s instructions with modifications that included an optimized DNase treatment step (FavorPrep Blood/Cultured Cell Total RNA Purification Mini Kit). Next, extracted RNA (500 ng) was used as the template for reverse transcription to cDNA. Reverse transcription quantitative PCR (RT-qPCR) was performed using the 2× iQ SYBR Green supermix (Bio-Rad, Taiwan) in a StepOnePlus thermocycler system (Thermo Fisher Scientific Inc., USA). The primer pairs used for RT-qPCR are shown in Table S1, with *gyrA* as the housekeeping control gene. Quantification was performed using the 2^−ΔΔCt^ method (65). The ΔCt was obtained by subtracting the *gyrA* Ct value from the Ct value of the target genes. The fold change was calculated according to the formula 2^−ΔΔCt^, where ΔΔCt was the difference between ΔCt and the ΔCt calibrator value (which was assigned a value of 1 arbitrary unit). Gene expression profiling was performed in biological triplicate.

### Statistical analysis

Statistical analyses were performed using GraphPad Prism software (version 8.0.2; GraphPad Software, Inc., San Diego, CA). The data are expressed as mean ± SD. Differences between group means were analyzed using one-way ANOVA with Dunnett’s multiple comparisons test and unpaired t-tests (two-tailed) to find group differences. *P*-value < 0.05 was considered statistically significant.

## Data Availability

All data generated or analyzed during this study are included in this published article or in the supplement.

## SUPPLEMENTAL MATERIAL

Supplemental material aac.00199-25-s0001.docx Fig. S1 to S11; Tables S1 to S4.

ASM does not own the copyrights to Supplemental Material that may be linked to, or accessed through, an article. The authors have granted ASM a non-exclusive, world-wide license to publish the Supplemental Material files. Please contact the corresponding author directly for reuse.
