# Supplementary material for: Indolenine-substituted pyrazole derivative 4e inhibits planktonic Staphylococcus lugdunensis growth and biofilm formation by disrupting purine biosynthesis and compromising cell wall and membrane integrity
Source: Antimicrob Agents Chemother. 2025 Jul 23;69(9):e00199-25. doi: 10.1128/aac.00199-25 (PMC12406677; doi:10.1128/aac.00199-25)
Supplement: Supplemental material — Fig. S1 to S11; Tables S1 to S4. [file aac.00199-25-s0001.docx]

| **Gene** | **Primer sequence (5'-3')** | **Product size (bp)** | **Reference** |
| --- | --- | --- | --- |
| *purA* | AGGTGTAGGTCCAACCTTCG | 120 | This study |
|  | CGTCCTGTCGTCGTACCATA |  |  |
| *purB* | GCGGTGCTGTTGGTACATTT | 122 | This study |
|  | ATATGCGTGACGATCCCGTT |  |  |
| *guaA* | GAACGTGCGAACCAAAGAGA | 155 | This study |
|  | GGGCTGTCAGCAATCACTTC |  |  |
| *guaB* | AACACCTACGCCAGCAACTA | 113 | This study |
|  | TTGCAACAGCAGAAGCAACT |  |  |
| *murA* | CCAGGTGGTTGTGCTATTGG | 162 | This study |
|  | AGCTCCTACGCTTGGAAAGT |  |  |
| *murB* | CGCTATGCAAGACAGCATGA | 163 | This study |
|  | TAATCGCTGCACCACTACCA |  |  |
| *murC* | GAGGTTGTTAGAGCGCATGA | 196 | This study |
|  | TTCCCAAACCTGTGCCATCT |  |  |
| *murD* | AGTATCGTCCACACATCGCT | 160 | This study |
|  | TCAATCAAATGGCGTTGGTGA |  |  |
| *gyrA* | CGTGCACGCGCAGAAATTGA | 176 | This study |
|  | ACACCGGTGCGTAAGCTTGT |  |  |

**Table S1. Primers used in RT-qPCR**

**Table S2. Antibacterial activity of 4e against 22 clinical *S. lugdunensis* isolates with their antibiotic susceptibility, *mecA*, SCC*mec* types, and MLST.**

| **Strain** | **Specimen** | **4e (μg/mL)** | **Antibiotic susceptibility testing** | | | | | | | ***mecA* typing** | **SCC*mec* type** | **MLST** |
| --- | --- | --- | --- | --- | --- | --- | --- | --- | --- | --- | --- | --- |
|  |  |  | P | OX | CC | E | SXT | TEC | VAN |  |  |  |
| CGMH-SL20 | Blood | 62.5 | R | R | S | S | S | S | S | (+) | V | 3 |
| CGMH-SL134 | CSF | 62.5 | R | R | S | S | S | S | S | (+) | V | 3 |
| CGMH-SL104 | Blood | 62.5 | R | S | R | R | S | S | S | (+) | V | 3 |
| CGMH-SL138 | Blood | 62.5 | R | R | R | R | S | S | S | (+) | IV | 3 |
| CGMH-SL99 | Blood | 62.5 | R | S | S | S | S | S | S | (+) | V | 3 |
| CGMH-SL61 | Blood | 62.5 | R | R | S | S | S | S | S | (+) | V | 3 |
| CGMH-SL144 | Blood | 62.5 | R | R | S | S | S | S | S | (+) | VT | 3 |
| CGMH-SL49 | Blood | 62.5 | S | S | R | R | S | S | S | (-) | (-) | 3 |
| CGMH-SL33 | Blood | 62.5 | S | S | S | S | S | S | S | (-) | (-) | 3 |
| CGMH-SL19 | Blood | 31.25 | R | R | S | S | S | S | S | (+) | V | 3 |
| **CGMH-SL131** | **Blood** | **62.5** | **R** | **R** | **S** | **S** | **S** | **S** | **S** | **(+)** | **V** | **3** |
| **CGMH-SL11** | **Blood** | **62.5** | **S** | **S** | **S** | **S** | **S** | **S** | **S** | **(-)** | **(-)** | **6** |
| CGMH-SL36 | Blood | 62.5 | R | R | R | R | S | S | S | (+) | Untypable | 6 |
| CGMH-SL52 | Blood | 62.5 | R | R | R | R | S | S | S | (+) | II | 6 |
| CGMH-SL118 | Blood | 62.5 | R | R | R | R | S | S | S | (+) | II | 6 |
| CGMH-SL139 | Blood | 62.5 | R | R | R | R | S | S | S | (+) | II | 6 |
| CGMH-SL190 | Wound | 62.5 | R | R | R | R | S | S | S | (+) | II | 6 |
| CGMH-SL864 | Blood | 62.5 | R | R | R | R | S | S | S | (+) | Untypable | 6 |
| CGMH-SL868 | Blood | 62.5 | R | R | R | R | S | S | S | (+) | Untypable | 6 |
| CGMH-SL872 | Blood | 62.5 | R | R | R | R | S | S | S | (+) | II | 6 |
| CGMH-SL879 | Blood | 62.5 | R | R | R | R | S | S | S | (+) | II | 6 |
| CGMH-SL880 | Blood | 62.5 | R | R | R | R | S | S | S | (+) | II | 6 |

**Table S3A. Features with significant difference in supernatant for amide column.**

*: fold change (log2), ns, no significance.

| Alignment ID | Retention time (min) | Detected *m/z* | Theoretical *m/z* | Mass error  (ppm) | Adduct type | Formula | Metabolite name | 31.25/DMSO* | 62.5/DMSO* | 31.25/62.5* |
| --- | --- | --- | --- | --- | --- | --- | --- | --- | --- | --- |
| 327 | 3.39 | 162.1137 | 162.113 | 4.56 | [M+H]+ | C7H16NO3 | Carnitine | -1.08 | ns | ns |
| 1514 | 3.38 | 371.2339 | 371.2335 | 1.14 | [M+H]+ | C22H30N2O3 | unknown | -2.38 | ns | ns |
| 1576 | 1.01 | 382.1537 | 382.1556 | -4.87 | [M+H]+ | C24H19N3O2 | 4e | 13.44 | ns | ns |

|  | Retention time (min) | Detected *m/z* | Theoretical *m/z* | Mass error(ppm) | Adduct type | Formula | Metabolite name | 31.25/DMSO* | 6.25/DMSO* | 31.25/6.25* |
| --- | --- | --- | --- | --- | --- | --- | --- | --- | --- | --- |
| 42 | 3.14 | 86.0958 | 86.0964 | -7.2 | [M+H]+ | C5H11N | Piperidine | -2.65 | -1.07 | -1.58 |
| 201 | 4.73 | 133.0606 | 133.0608 | -1.43 | [M+H]+ | C4H8N2O3 | Asparagine | 1.4 | ns | 1.87 |
| 246 | 4.65 | 144.0992 | 144.1019 | -18.88 | [M+H]+ | C7H13NO2 | Proline betaine | ns | -1.33 | ns |
| 312 | 4.56 | 159.0781 | 159.0764 | 10.37 | [M+H]+ | C6H10N2O3 | hydroxyectoine | 1.06 | ns | ns |
| 322 | 4.65 | 161.1255 | 161.1285 | -18.87 | [M+NH4]+ | C7H13NO2 | Proline betaine | ns | -1.29 | ns |
| 343 | 3.04 | 166.0836 | 166.0868 | -19.17 | [M+H]+ | C9H11NO2 | Phenylalanine | -2.18 | -1.05 | -1.13 |
| 380 | 5.38 | 175.1219 | 175.1195 | 13.87 | [M+H]+ | C6H14N4O2 | Arginine | 1.89 | ns | 1.47 |
| 384 | 4.8 | 176.1036 | 176.103 | 3.12 | [M+H]+ | C6H13N3O3 | Citrulline | 1.28 | ns | ns |
| 388 | 3.14 | 178.0863 | 178.0863 | 0.17 | [M+NH4]+ | C10H8O2 | Naphthalene-1,2-diol | 2.59 | 2.7 | ns |
| 527 | 4.52 | 205.0499 | 205.0495 | 2.15 | [M+H]+ | C11H8O4 | 1,4-Dihydroxy-2-naphthoic acid | ns | -1.03 | ns |
| 697 | 5.13 | 236.1527 | 236.1512 | 6.14 | [M+H-H2O]+ | C11H19N5O2 | Cyclo(L-Arg-D-Pro) | 1.06 | ns | ns |
| 963 | 4.91 | 276.1552 | 276.1554 | -0.8 | [M+H]+ | C11H20N2O6 | epsilon-(gamma-Glutamyl)lysine | 5.05 | ns | 5.67 |
| 1001 | 3.89 | 283.9954 | 283.9931 | 8.17 | [M+NH4]+ | C3H3O10P2 | (2R)-2,3-bisphosphoglyceric acid | -1.04 | ns | ns |
| 1012 | 3.09 | 285.1704 | 285.1704 | -0.07 | [M+NH4-H2O]+ | C16H19N3O2 | L-valyl-L-tryptophan anhydride | -3.74 | -1.71 | -2.ns 02 |
| 1047 | 5.93 | 291.1314 | 291.1299 | 5.08 | [M+H]+ | C10H18N4O6 | Argininosuccinic acid | 1.04 | 1.47 |  |
| 1180 | 3.64 | 314.1264 | 314.1293 | -9.25 | [M+H]+ | C20H15N3O | Angustine | 2.58 | ns | 1.81 |

**Table S3B. Features with significant difference in pellet for amide column.**

| Alignment ID | Retention time (min) | Detected *m/z* | Theoretical *m/z* | Mass error  (ppm) | Adduct type | Formula | Metabolite name | 31.25/DMSO* | 6.25/DMSO* | 31.25/6.25* |
| --- | --- | --- | --- | --- | --- | --- | --- | --- | --- | --- |
| 1207 | 3.91 | 318.1198 | 318.1173 | 7.7 | [M+Na]+ | C12H17N5O4 | N6,N6-Dimethyladenosine | -3.52 | -2.14 | ns |
| 1235 | 4.79 | 323.1435 | 323.1449 | -4.36 | [2M+H]+ | C6H11NO4 | N-methyl-L-glutamic Acid | 1.32 | ns | ns |
| 1263 | 3.9 | 328.0275 | 328.0305 | -9.05 | [M+Na]+ | C9H12N3O7P | Cytidine 2',3'-cyclic phosphate | -1.47 | ns | ns |
| 1288 | 4.6 | 331.224 | 331.2201 | 11.68 | [M+H]+ | C12H26N8O3 | Arginyl-L-arginine | 3.14 | 4.26 | -1.12 |
| 1315 | 5.27 | 337.0246 | 337.0324 | -23.22 | [M+Na]+ | C16H10O7 | unknown | -5.19 | -1.87 | ns |
| 1382 | 5.1 | 348.0727 | 348.0704 | 6.49 | [M+H]+ | C10H14N5O7P | Adenosine monophosphate (AMP) | -1.83 | -1 | ns |
| 1477 | 5.53 | 364.0676 | 364.0653 | 6.24 | [M+H]+ | C10H14N5O8P | Guanosine monophosphate | -3.18 | ns | ns |
| 1527 | 6.21 | 373.6837 | 373.684 | -0.94 | [M+2H]2+ | C36H51N5O12 | unknown | 6.37 | 3.33 | 3.04 |
| 1558 | 4.46 | 377.2197 | 377.217 | 7.08 | [2M+H]+ | C9H16O4 | Azelaic acid | 1.22 | 2.88 | -1.66 |
| 1576 | 1.01 | 382.1537 | 382.1556 | -4.87 | [M+H]+ | C24H19N3O2 | 4e | 4.01 | 2.83 | 1.18 |
| 1668 | 5.73 | 399.1262 | 399.1275 | -3.28 | [M+Na]+ | C17H20N4O6 | Riboflavin | -1.94 | ns | ns |
| 1697 | 6.21 | 405.0109 | 405.0095 | 3.36 | [M+H]+ | C9H14N2O12P2 | Uridine 3',5'-phosphate | 4.46 | ns | 2.83 |
| 1699 | 4.32 | 405.2458 | 405.2483 | -6.27 | [2M+H]+ | C10H18O4 | Sebacic acid | ns | 5.26 | -1.9 |
| 1778 | 6.21 | 426.9925 | 426.9914 | 2.55 | [M+Na]+ | C9H14N2O12P2 | Uridine 3',5'-phosphate | 4.46 | ns | 2.84 |
| 1785 | 5.65 | 428.0174 | 428.0272 | -22.99 | [M+H]+ | C10H14N5O10PS | Adenosine phosphosulfate | -1.02 | ns | ns |
| 1802 | 4.01 | 433.231 | 433.2221 | 20.52 | [M+H]+ | C24H32O7 | unknown | 7.11 | ns | 2.64 |
| 1862 | 4.32 | 449.1841 | 449.1918 | -17.05 | [M+NH4]+ | C22H25NO8 | unknown | 3.83 | ns | 2.64 |
| 1895 | 4.6 | 461.1794 | 461.1772 | 4.73 | [M+H-H2O]+ | C19H30N2O12 | N-Acetyl-D-glucosamine(anhydrous)N-Acetylmuramic acid | 1.55 | 1.3 | ns |
| 2047 | 4.7 | 515.2147 | 515.2129 | 3.42 | [2M+H]+ | C8H20NO6P | Glycerophosphocholine | 1.14 | 1.8 | ns |
| 2057 | 3.26 | 521.1981 | 521.2023 | -8.14 | [M-H2O+H]+ | C26H34O12 | unknown | -3.85 | -2.27 | ns |

**Table S3B. (continued)**

*: fold change (log2), ns, no significance.

**Table S3B. (continued)**

| Alignment ID | Retention time (min) | Detected *m/z* | Theoretical *m/z* | Mass error  (ppm) | Adduct  type | Formula | Metabolite name | 31.25/DMSO* | 6.25/DMSO* | 31.25/6.25* |
| --- | --- | --- | --- | --- | --- | --- | --- | --- | --- | --- |
| 2047 | 4.7 | 515.2147 | 515.2129 | 3.42 | [2M+H]+ | C8H20NO6P | Glycerophosphocholine | 1.14 | 1.8 | ns |
| 2057 | 3.26 | 521.1981 | 521.2023 | -8.14 | [M-H2O+H]+ | C26H34O12 | unknown | -3.85 | -2.27 | ns |
| 2061 | 4.7 | 523.2005 | 523.1977 | 5.35 | [2M+H-H2O]+ | C15H13N2O3 | cyclo-acetoacetyl-L-tryptophan | ns | 1.31 | ns |
| 2095 | 4.68 | 537.1669 | 537.1688 | -3.59 | [2M+H]+ | C10H12N4O5 | Inosine | 1.64 | 2.07 | ns |
| 2099 | 3.26 | 539.2101 | 539.2123 | -4.12 | [M+H]+ | C26H34O12 | unknown | -4.44 | -2.5 | ns |
| 2104 | 4.7 | 541.2095 | 541.2082 | 2.35 | [2M+H]+ | C15H13N2O3 | cyclo-acetoacetyl-L-tryptophan | 1.87 | 2.39 | ns |
| 2160 | 4.46 | 565.3263 | 565.322 | 7.63 | [4M+H]+ | C9H16O4 | Azelaic acid | 2.79 | 4.98 | -2.19 |
| 2182 | 6.21 | 575.6853 | 575.6831 | 3.82 | [M+2H]2+ | C40H65N9O26P2 | UDP-N-Acetylmuramoyl-L-alanyl-D-glutamyl-L-lysyl-D-alanyl-D-alanine | 5.49 | 2.8 | 2.69 |
| 2203 | 3.91 | 583.1442 | 583.1428 | 2.35 | [2M+H]+ | C13H13N3O3S | unknown | -5 | -3.72 | ns |
| 2212 | 6.2 | 586.666 | 586.674 | -13.72 | [M+H+Na]2+ | C40H65N9O26P2 | UDP-N-Acetylmuramoyl-L-alanyl-D-glutamyl-L-lysyl-D-alanyl-D-alanine | 5.47 | 2.96 | 2.51 |
| 2232 | 6.2 | 597.6317 |  |  |  |  | unknown | 5.61 | ns | 3.42 |
| 2244 | 6.25 | 604.1937 |  |  |  |  | unknown | 4.93 | ns | 3.35 |
| 2247 | 4.6 | 607.2436 | 607.2527 | -15 | [M+Na]+ | C33H36N4O6 | Bilirubin | 2.05 | ns | ns |
| 2386 | 5.71 | 702.0902 | 702.0919 | -2.41 | [M+Na]+ | C20H31N3O19P2 | UDP-N-acetylmuramate | 2.04 | ns | 1.92 |
| 2421 | 4.8 | 736.2419 | 736.2319 | 13.64 | [M+H]+ | C30H35N9O12 | N5-methyl--tetrahydropteroyl tri-L-glutamate | -1.09 | ns | ns |
| 2430 | 6.2 | 746.3574 | 746.3607 | -4.48 | [M+H]+ | C36H51N5O12 | unknown | 5.69 | ns | 3.19 |
| 2432 | 5.67 | 751.1476 | 751.1476 | -0.06 | [M+H]+ | C9H17N4O9P | UDP-N-acetylmuramoyl-L-alanine | 6.38 | ns | 4.59 |
| 2439 | 4.46 | 753.4359 | 753.4269 | 11.91 | [3M+H]+ | C9H16O4 | Azelaic acid | ns | 4.7 | -4.21 |
| 2446 | 5.36 | 764.3671 |  |  |  |  | unknown | 6.49 | ns | 2.59 |
| 2485 | 2.82 | 851.6152 | 851.6096 | 6.63 | [M+H]+ | C45H86O14 | unknown | -2.42 | -1.99 | ns |

*: fold change (log2), ns, no significance.

**Table S4A. Features with significant difference in supernatant for C18 column.**

| Alignment ID | Retention time (min) | Detected *m/z* | Theoretical *m/z* | Mass error | Adduct type | Formula | Metabolite name | 31.25/DMSO* | 6.25/DMSO* | 31.25/6.25* |
| --- | --- | --- | --- | --- | --- | --- | --- | --- | --- | --- |
| 577 | 4.15 | 266.1156 | 266.1181 | -9.41 | [M+H]+ | C17H15NO2 | 4e-related product | 8.23 | 6.2 | 2.03 |
| 924 | 5.07 | 338.1671 | 338.1657 | 4.1 | [M+H]+ | C23H19N3 | 4e-related product | 3.29 | ns | 2.45 |
| 1071 | 4.71 | 367.1321 | 367.1321 | 0.01 | [M+H]+ | C23H16N3O2 | 4e-related product | 8.52 | 7.48 | 1.05 |
| 1132 | 4.72 | 382.1594 | 382.1556 | 10.12 | [M+H]+ | C24H19N3O2 | 4e | 8.93 | 8.43 | ns |
| 1196 | 5.08 | 396.1708 | 396.1712 | -1.14 | [M+H]+ | C25H21N3O2 | 4e-related product | 2.3 | ns | 2.29 |
| 1271 | 4.55 | 415.2116 | 415.2134 | -4.46 | [M+H]+ | C25H27N4O2 | unknown | 2.7 | 1.76 | ns |

*: fold change (log2), ns, no significance.

| Alignment ID | Retention time (min) | Detected *m/z* | Theoretical *m/z* | Mass error | Adduct type | Formula | Metabolite name | 31.25/DMSO* | 62.5/DMSO* | 31.25/62.5* |
| --- | --- | --- | --- | --- | --- | --- | --- | --- | --- | --- |
| 46 | 1.37 | 103.0542 | 103.0548 | -6.31 | [M+H]+ | C8H6 | Phenylalanine (fragment) | -1.08 | ns | ns |
| 87 | 1.38 | 120.0816 | 120.0802 | 11.99 | [M+H]+ | C8H9N | Phenylalanine (fragment) | -1.37 | ns | ns |
| 93 | 1.38 | 126.0923 | 126.0913 | 7.53 | [M+NH4]+ | C7H8O | Benzyl alcohol | ns | 1.64 | -1.17 |
| 148 | 1.53 | 150.0591 | 150.0583 | 5.2 | [M+H]+ | C5H11NO2S | Methionine | -1.24 | ns | -1.39 |
| 157 | 0.54 | 156.0745 | 156.0768 | -14.61 | [M+H]+ | C6H9N3O2 | Histidine | -1.44 | -1.61 | ns |
| 182 | 1.38 | 166.0875 | 166.0863 | 7.1 | [M+H]+ | C9H11NO2 | Phenylalanine | -1.18 | ns | ns |
| 232 | 2.19 | 183.0922 | 183.0917 | 2.51 | [M+H]+ | C12H10N2 | Harman | 1.93 | ns | 1.94 |
| 264 | 1.79 | 192.0696 | 192.0689 | 3.85 | [M+H]+ | C7H13NO3S | N-Acetyl-L-methionine | -3.65 | ns | -3.8 |
| 276 | 1.39 | 196.0766 | 196.075 | 8.16 | [M+NH4]+ | C5H10N2O3S | Glycyl-Cysteine | -1.36 | -1.06 | ns |
| 353 | 0.96 | 217.1325 | 217.1295 | 13.91 | [M+H]+ | C8H16N4O3 | N-a-Acetyl-L-arginine | 3.34 | 2.85 | ns |
| 429 | 1.42 | 231.1702 | 231.1703 | -0.52 | [M+H]+ | C11H22N2O3 | Valylleucine | 2.35 | 2.17 | ns |
| 577 | 4.15 | 266.1156 | 266.1181 | -9.41 | [M+H]+ | C17H15NO2 | 4e-related product | 4.59 | 2.52 | 2.07 |
| 767 | 1.91 | 302.2012 | 302.1962 | 16.64 | [M+NH4]+ | C15H24O5 | unknown | 6.02 | ns | 5.45 |
| 924 | 5.07 | 338.1671 | 338.1657 | 4.1 | [M+H]+ | C23H19N3 | 4e-related product | 7.84 | 5.09 | 2.75 |
| 989 | 1.17 | 348.0715 | 348.0704 | 3.16 | [M+H]+ | C10H14N5O7P | Adenosine monophosphate (AMP) | -1.87 | ns | ns |
| 1071 | 4.71 | 367.1321 | 367.1321 | 0.01 | [M+H]+ | C23H16N3O2 | 4e-related product | 4.75 | 2.86 | 1.89 |
| 1100 | 1.33 | 373.6822 | 373.684 | -4.92 | [M+2H]2+ | C36H51N5O12 | unknown | 5.36 | 2.45 | 2.91 |
| 1132 | 4.72 | 382.1594 | 382.1556 | 10.12 | [M+H]+ | C24H19N3O2 | 4e | 4.5 | 3.3 | 1.2 |
| 1196 | 5.08 | 396.1708 | 396.1712 | -1.14 | [M+H]+ | C25H21N3O2 | 4e-related product | 7.44 | 5.03 | 2.41 |
| 1234 | 1.33 | 405.0105 | 405.0095 | 2.54 | [M+H]+ | C9H14N2O12P2 | Uridine 3',5'-phosphate (UDP) | 4.4 | 1.59 | 2.81 |
| 1546 | 1.38 | 521.1982 | 521.1991 | -1.77 | [2M+H-H2O]+ | C11H15N3O5 | N4-acetyl-2'-deoxycytidine | -3.57 | -1.82 | ns |
| 1549 | 6.54 | 523.4722 | 523.4721 | 0.13 | [M+H-H2O]+ | C33H64O5 | DG 30:0 (PG fragment) | -1.29 | ns | ns |
| 1578 | 6.61 | 537.4883 | 537.4877 | 1.19 | [M+H-H2O]+ | C34H66O5 | DG 31:0 (PG fragment) | -1.24 | ns | ns |

**Table S4B. Features with significant difference in pellet for C18 column.**

| Alignment ID | Retention time (min) | Detected *m/z* | Theoretical *m/z* | Mass error | Adduct type | Formula | Metabolite name | 31.25/DMSO* | 62.5/DMSO* | 31.25/62.5* |
| --- | --- | --- | --- | --- | --- | --- | --- | --- | --- | --- |
| 1582 | 1.38 | 539.2098 | 539.2096 | 0.45 | [2M+H]+ | C11H15N3O5 | N4-acetyl-2'-deoxycytidine | -3.74 | -2.02 | ns |
| 1604 | 6.65 | 551.5059 | 551.5034 | 4.57 | [M+H-H2O]+ | C35H68O5 | DG 32:0 (PG fragment) | -1.51 | ns | ns |
| 1637 | 6.73 | 565.5209 | 565.519 | 3.31 | [M+H-H2O]+ | C36H70O5 | DG 33:0 (PG fragment) | -1.2 | ns | ns |
| 1664 | 6.77 | 579.5381 | 579.5347 | 5.85 | [M+H-H2O]+ | C37H72O5 | DG 34:0 | -1.03 | ns | ns |
| 1748 | 6.89 | 621.5827 | 621.5816 | 1.77 | [M+H-H2O]+ | C40H78O5 | DG 37:0 | 1.11 | ns | ns |
| 1911 | 6.61 | 709.5026 | 709.5014 | 1.63 | [M+H]+ | C37H73O10P | PG 31:0 | -1.12 | ns | ns |
| 1913 | 6.54 | 712.5122 | 712.5123 | -0.21 | [M+NH4]+ | C36H71O10P | PG 30:0 | -1.56 | ns | ns |
| 1928 | 6.65 | 723.519 | 723.5171 | 2.6 | [M+H]+ | C38H75O10P | PG 32:0 | -1.31 | ns | ns |
| 1931 | 6.61 | 726.5286 | 726.528 | 0.77 | [M+NH4]+ | C37H73O10P | PG 31:0 | -1.06 | ns | ns |
| 1944 | 6.72 | 737.5343 | 737.5327 | 2.17 | [M+H]+ | C39H77O10P | PG 33:0 | -1.02 | ns | ns |
| 1945 | 6.55 | 738.5082 | 738.5068 | 1.95 | [M+H]+ | C41H72NO8P | PC 33:5 | -1.66 | -1.12 | ns |
| 1946 | 6.65 | 740.5443 | 740.5436 | 0.96 | [M+NH4]+ | C38H75O10P | PG 32:0 | -1.23 | ns | ns |
| 1954 | 1.33 | 746.3593 | 746.3607 | -1.94 | [M+H]+ | C36H51N5O12 | unknown | 4.91 | ns | 2.96 |
| 1962 | 6.86 | 748.593 | 748.5851 | 10.58 | [M+H]+ | C41H82NO8P | PC 33:0 | -1.18 | ns | ns |
| 1963 | 6.66 | 749.5122 | 749.5116 | 0.81 | [M+H]+ | C43H73O8P | PA 40:6 | -1.24 | ns | ns |
| 1979 | 6.72 | 763.5295 | 763.5272 | 3.06 | [M+H]+ | C44H75O8P | PA 41:6 | -1.03 | ns | ns |
| 1985 | 6.65 | 766.5414 | 766.5381 | 4.28 | [M+H]+ | C43H76NO8P | PC 35:5 | -1.27 | ns | ns |
| 2029 | 6.66 | 810.5511 | 810.5432 | 9.73 | [M+H]+ | C48H78NO8P | unknown | -1.38 | ns | ns |
| 2039 | 6.56 | 823.5797 | 823.5847 | -6.06 | [M+Na]+ | C52H80O6 | TG 49:10 | -1.38 | -1.48 | ns |
| 2058 | 6.68 | 851.6127 | 851.616 | -3.84 | [M+Na]+ | C54H84O6 | TG 51:10 | ns | -1.2 | ns |
| 2076 | 6.8 | 879.6487 | 879.6473 | 1.64 | [M+Na]+ | C56H88O6 | TG 53:10 | ns | -1.43 | ns |
| 2080 | 6.85 | 893.6651 | 893.663 | 2.35 | [M+Na]+ | C57H90O6 | TG 54:10 | -1.25 | -1.41 | ns |
| 2141 | 1.32 | 1150.3738 | 1150.3589 | 12.94 | [M+H]+ | C40H65N9O26P2 | UDP-N-Acetylmuramoyl-L-alanyl-gamma-D-glutamyl-L-lysyl-D-alanyl-D-alanine | 4.64 | ns | ns |

**Table S4B. (continued)**

*: fold change (log2), ns, no significance.

**Figures**

**Figure S1**


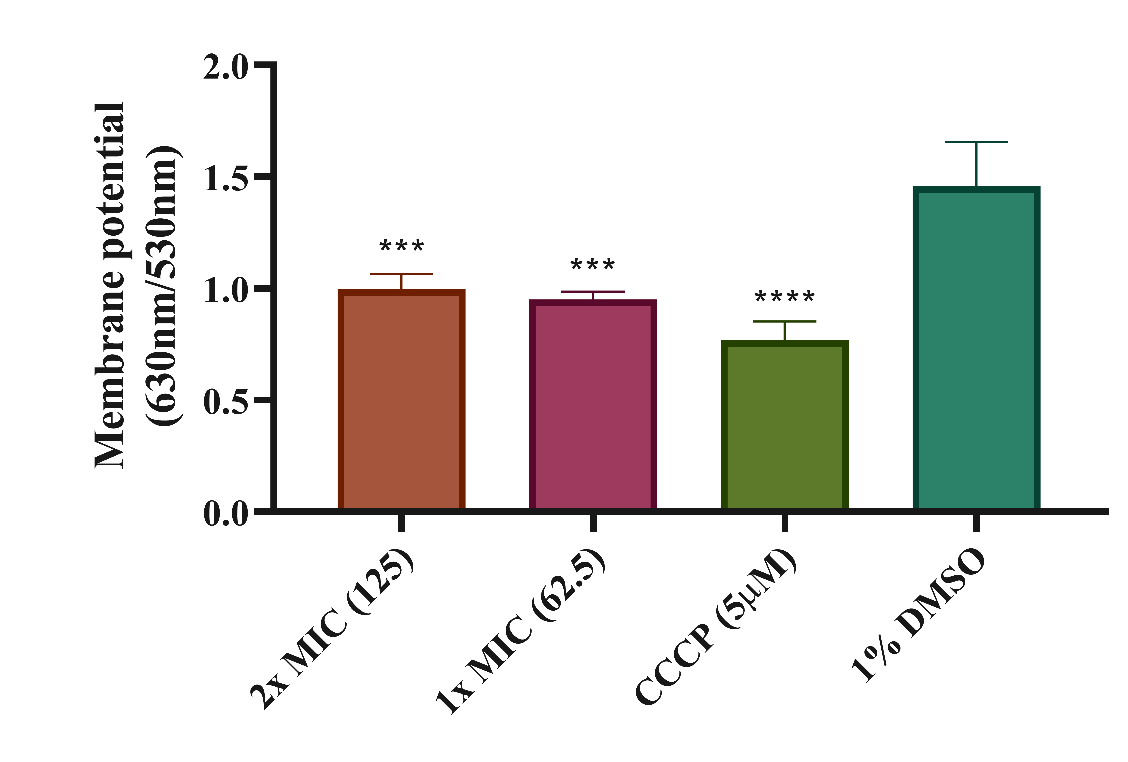


**Fig. S1. Effect of 4e to CGMH-SL131 determined using membrane potential assay.**  Membrane potential assay of 4e-treated cells (1x MIC and 2x MIC) at 24 h, conducted using a microplate reader. Negative and positive controls were 1% DMSO and CCCP (5 μM), respectively, with statistical analysis using 1% DMSO as the comparison control. The concentration of 4e (μg/mL) is indicated in parentheses. All experiments were performed in triplicate, with three biological and three technical replicates. ns, no significance; ***, *p*<0.001; ****, *p*<0.0001.

**Figure S2**

**Fig. S2. Biofilm formation of *S. lugdunensis* clinical isolates using 0.1% crystal violet staining**. All strains are strong biofilm-formers except for CGMH-SL33, which forms a moderate biofilm. The capability of the tested *S. lugdunensis* strains to biofilm formation was categorized as follows: OD ≤ OD cut-off (ODc), non-biofilm forming; ODc < OD ≤ 2 × ODc, weak biofilm-forming; 2 × ODc < OD ≤ 4 × ODc, moderate biofilm-forming; 4 × ODc < OD, strong biofilm-forming. ODc was considered as three standard deviations (SDs) above the mean OD of the negative control. TSB without bacterial cells was applied as the negative control. CGMH-SL131 and CGMH-SL11 were selected as test pathogens for the antibiofilm assays due to their differences in oxacillin susceptibility and multilocus sequence typing (MLST). All experiments were performed in three biological replicates, each with three technical replicates.

**Figure S3**

**(A).**


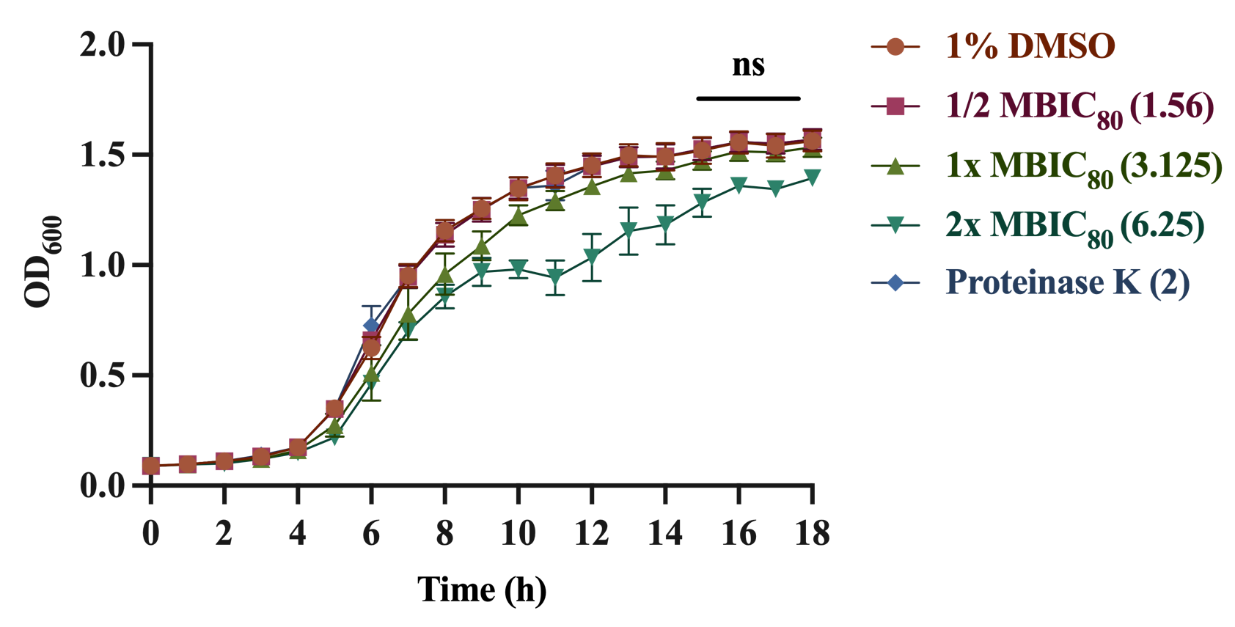


**(B).**


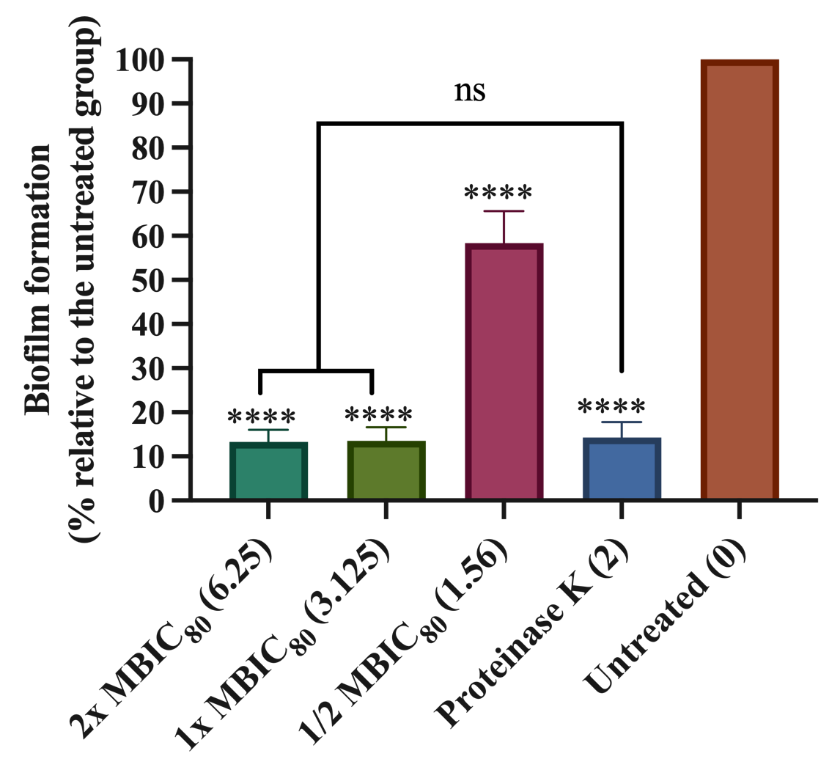


**(C).**


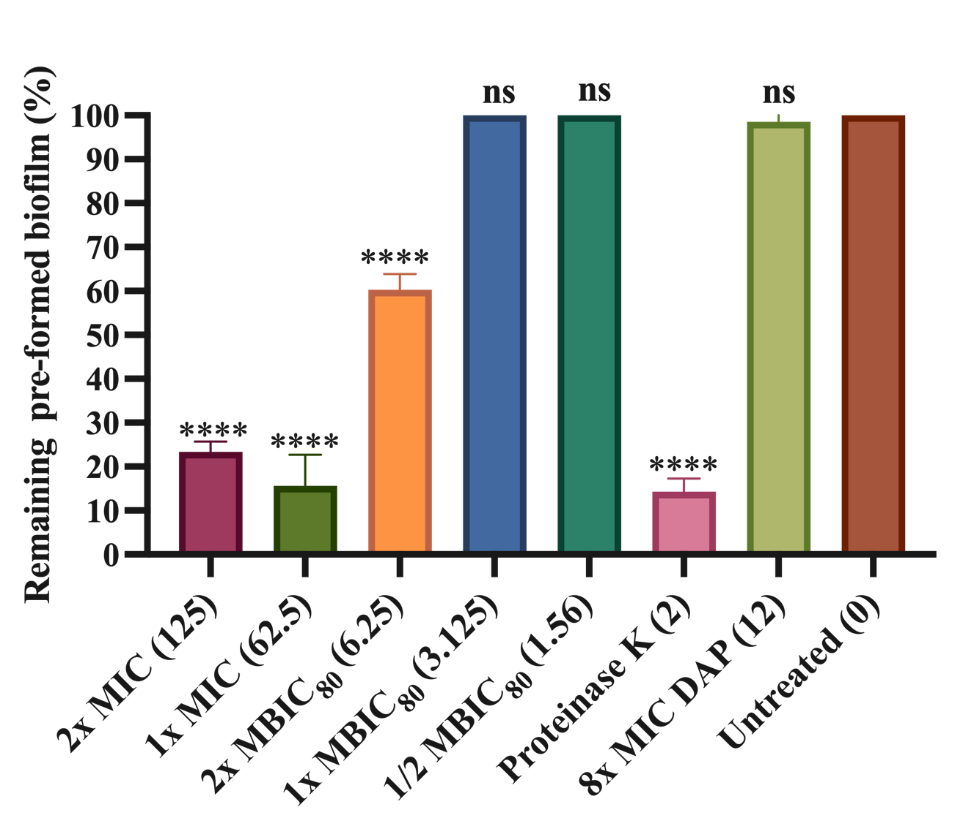


**(D).**


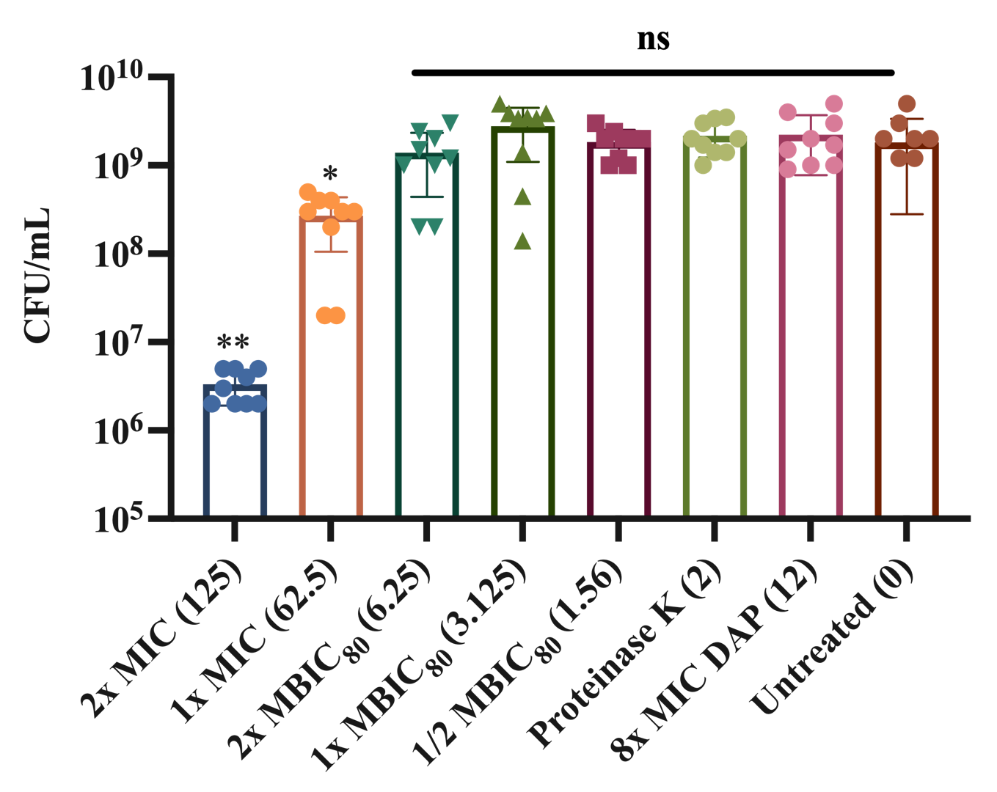


**Fig. S3. Antibiofilm activity of 4e against CGMH-SL11. (A)**. Growth curve of antibiofilm concentrations. **(B).** % Biofilm formation of CGMH-SL11 after 24 exposure to 4e at 1x MBIC_80_ (3.125 μg/mL) and 2x MBIC_80_ (6.25 μg/mL). **(C).** % remaining pre-formed biofilm after 4e treatment for 24 h. **(D).** Combined CFU counts of the bacterial cells in the planktonic and biofilm after 4e treatment for 24 h. The concentration (μg/mL) of 4e and proteinase K is indicated in parentheses. All experiments were performed in three biological replicates, each with three technical replicates. ns, no significance; *, *p*<0.002; **, *p*<0.0005 ****, *p*<0.0001.

**Figure S4**

**(A).**


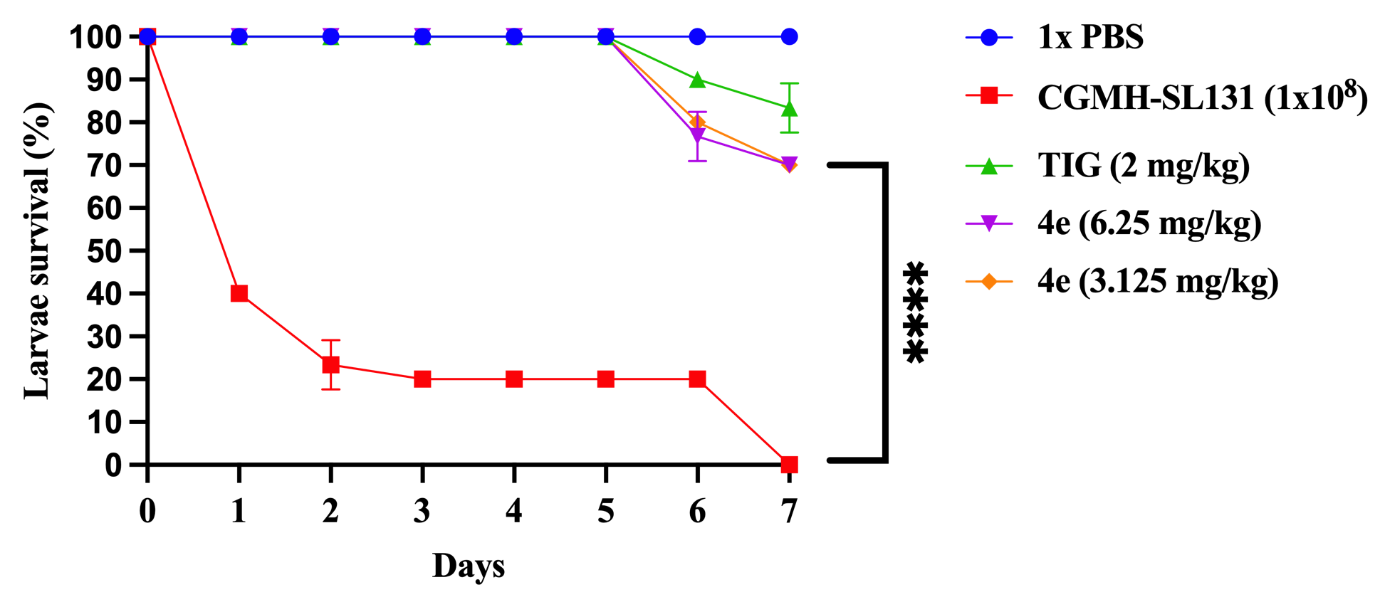


**(B).**


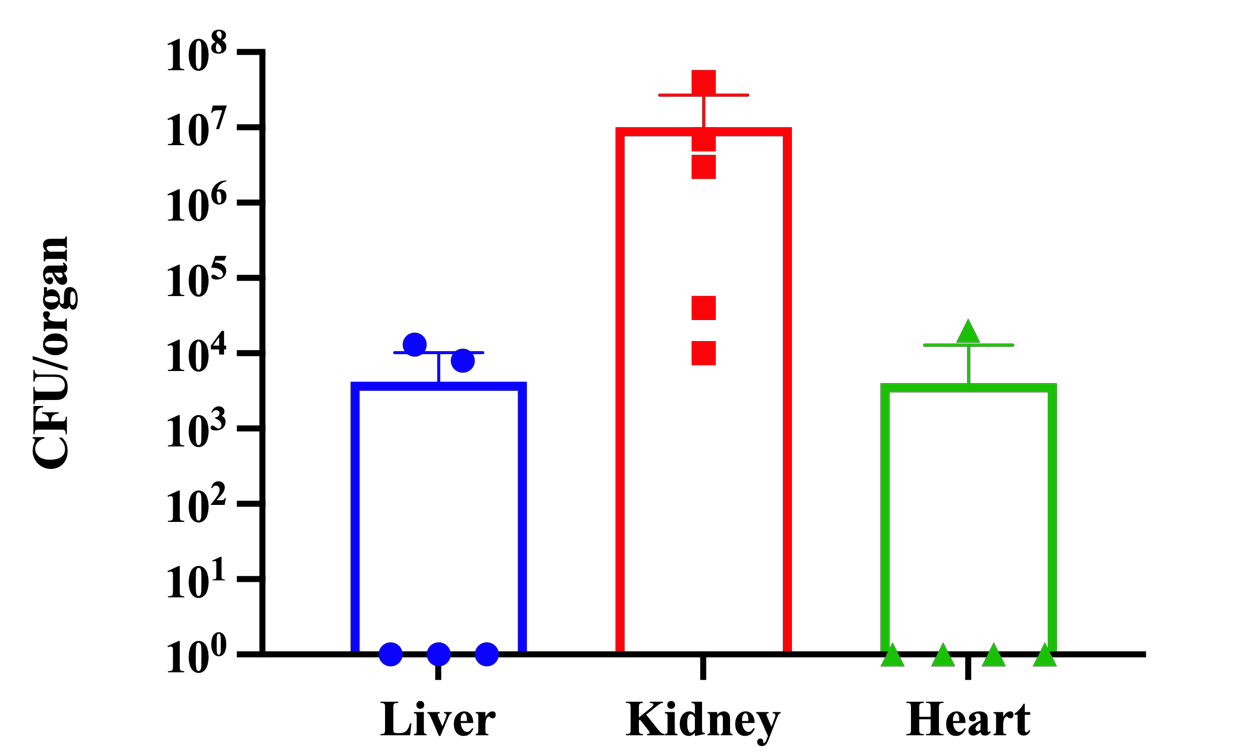


**Fig. S4. *In vivo* studies of CGMH-SL131 and 4e. (A).** The percentage of larvae survival treated with 4e (6.25 mg/kg and 3.125 mg/kg) alone. Larvae infected with high CFU (1 x 10^6^) of CGMH-SL131 were used as a lethal control. Ten larvae were injected in this assay and monitored for their survival for 7 days. **(B).** CGMH-SL131 burden to organs determined 72 hours post-infection. ****, *p*<0.0001.

**Figure S5**

**(A).**

**
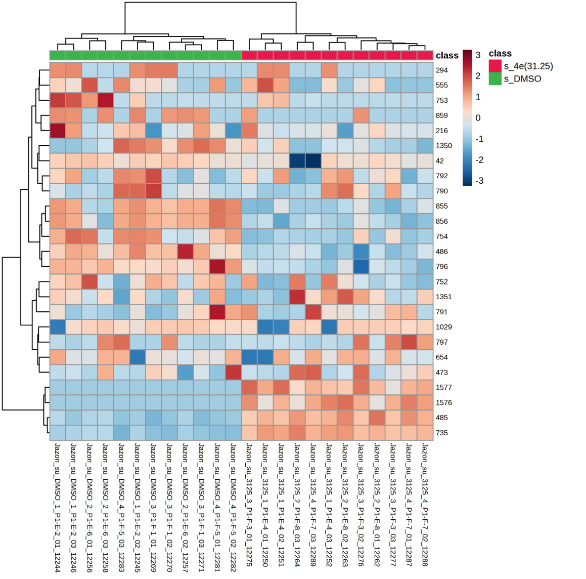
**

**(B).**

**
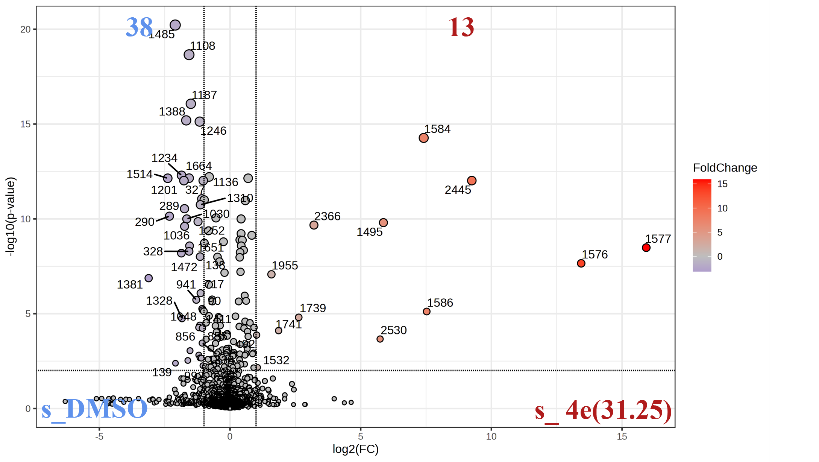
**

**(C).**

**
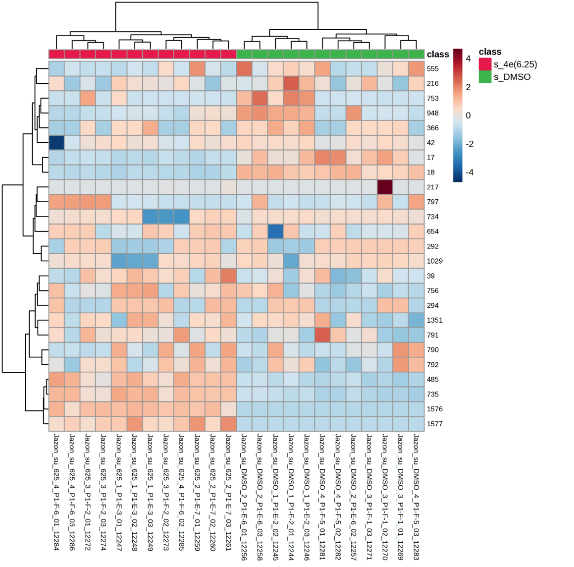
**

**(D).**


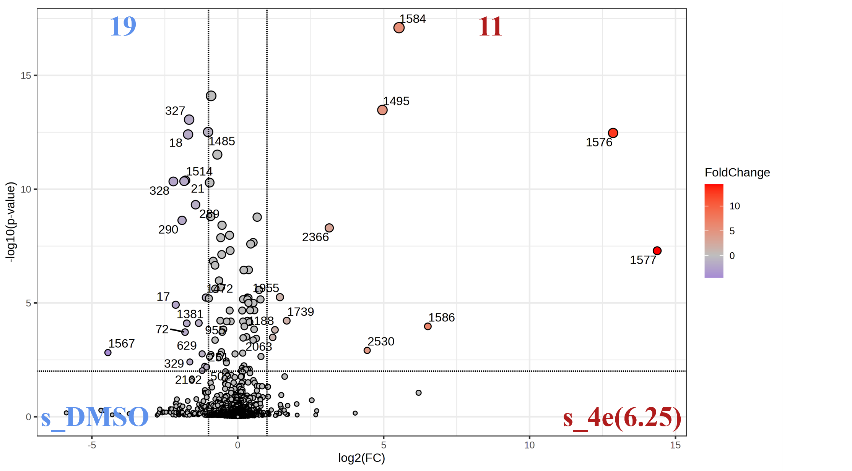


**Fig. S5. Metabolomics studies in supernatant (amide column).** **(A).** Heat map of the top 25 features obtained from the supernatant with highest scores from the PLS-DA and volcano plot **(B)** showing the number of differentially expressed features between 4e-treated groups (31.25 (μg/mL)) when compared to DMSO-untreated group. **(C).** Heat map of the top 25 features obtained from the supernatant with highest scores from the PLS-DA and volcano plot **(D)** showing the number of differentially expressed features between 4e-treated groups (6.25 (μg/mL)) when compared to DMSO-untreated group. Cut-off point of log2 fold-change ≤ -1.5, log2 fold-change ≥ 1.5; and false detection rate (FDR < 0.05) was used in this experiment.

**Figure S6**

**(A).**

**
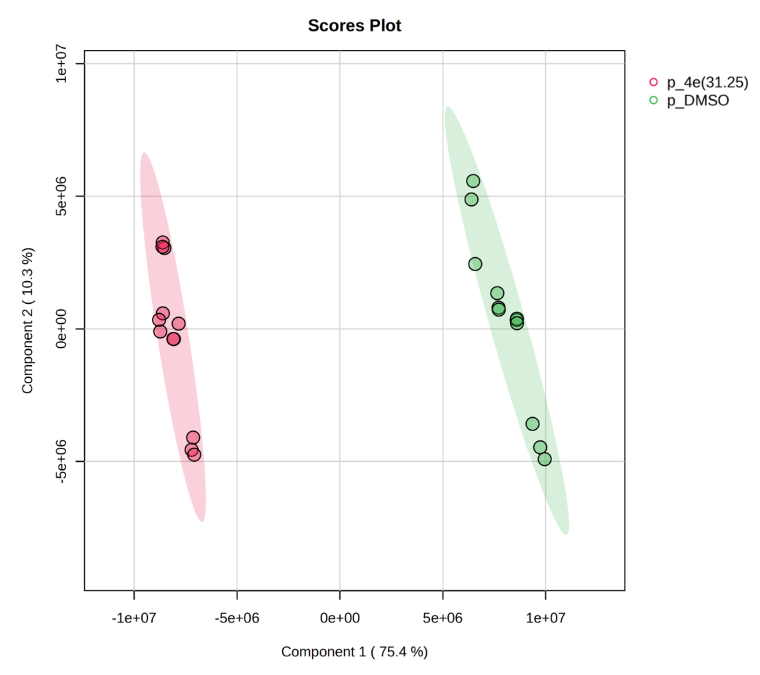
**

**(B).**

**
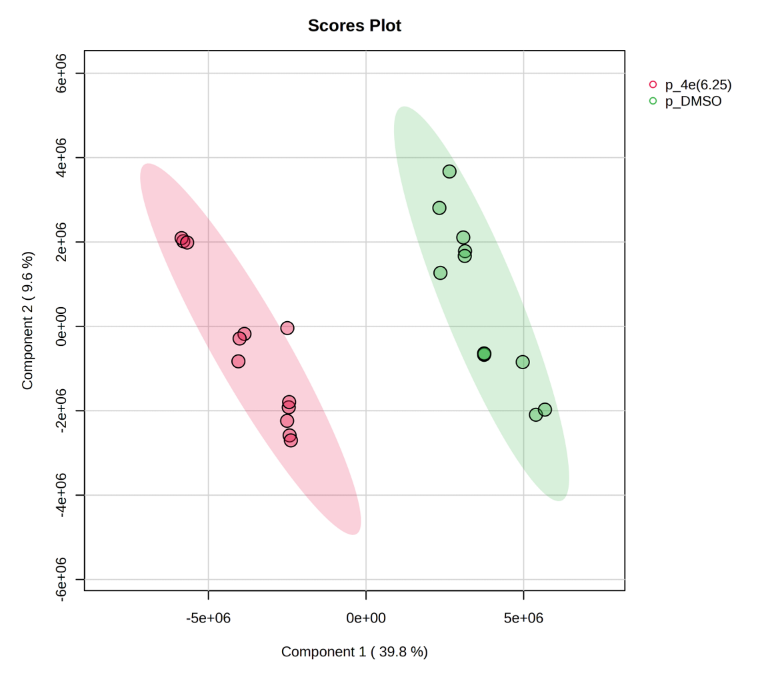
**

**(C).**

**
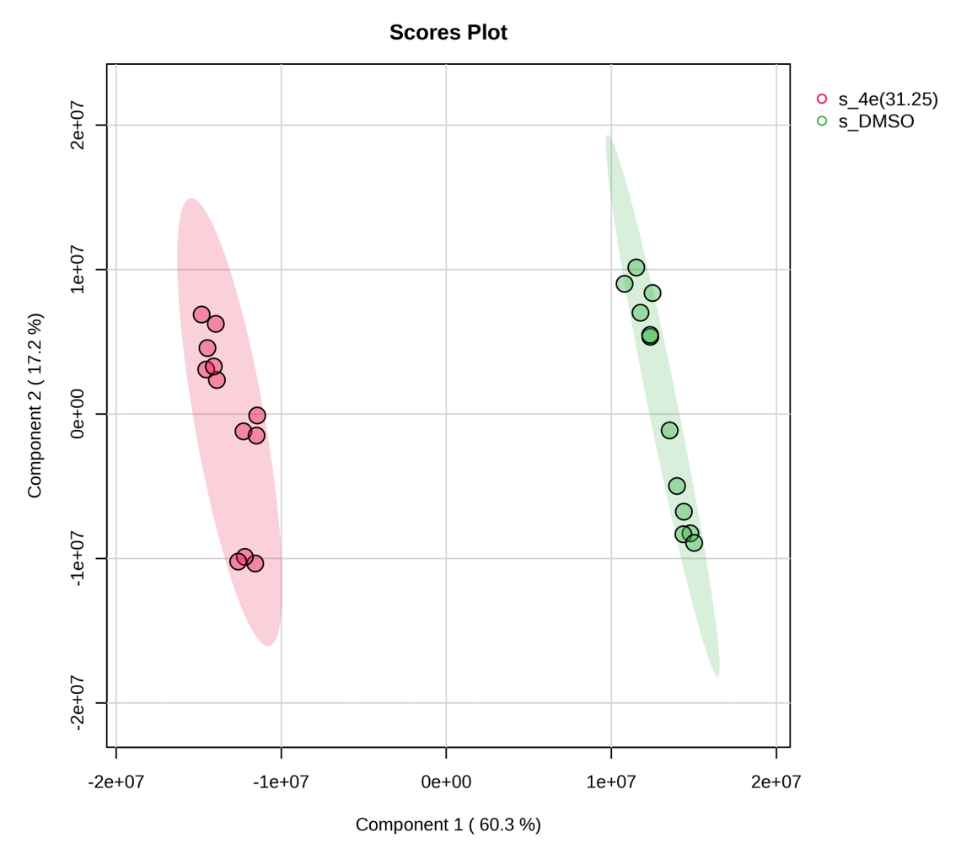
**

**(D).**

**
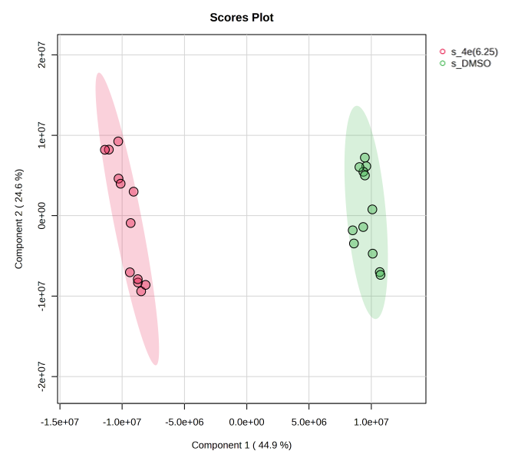
**

**Fig. S6. Metabolomics studies in supernatant and pellet (C18 column). (A, B)** PLS-DA of the features obtained from supernatant of 4e-treated groups (31.25 **(A)** and 6.25 **(B)** μg/mL) when compared to DMSO-untreated group. **(C, D)** PLS-DA of the features obtained from pellet of 4e-treated groups (31.25 **(C)** and 6.25 **(D)** μg/mL) when compared to DMSO-untreated group. Cut-off point of log2 fold-change ≤ -1.5, log2 fold-change ≥ 1.5; and false detection rate (FDR < 0.05) was used in this experiment.

**Figure S7**

**(A).**

**
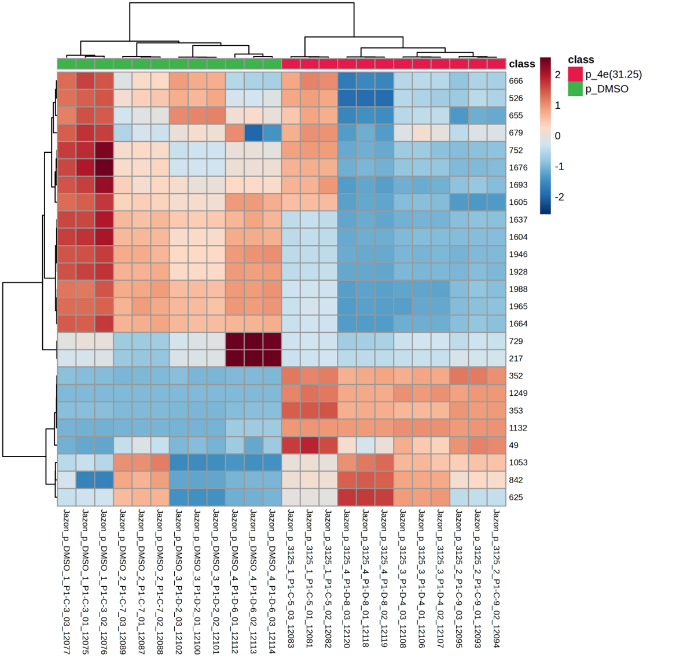
**

**(B).**

**
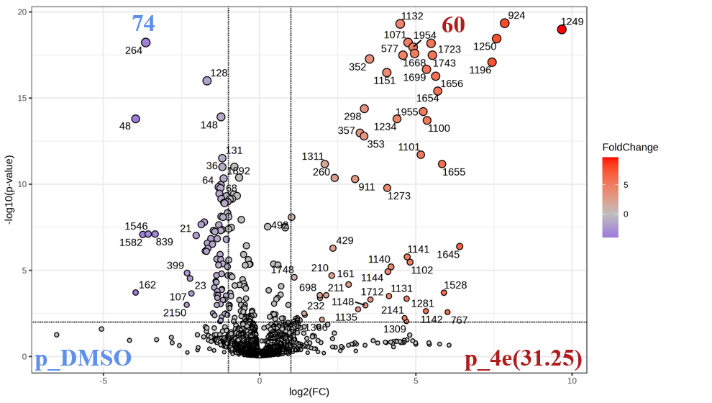
**

**(C).**

**
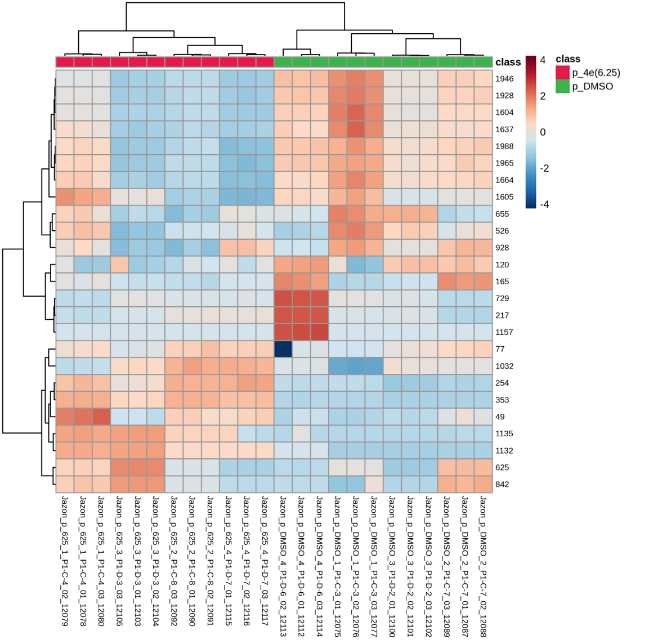
**

**(D).**

**
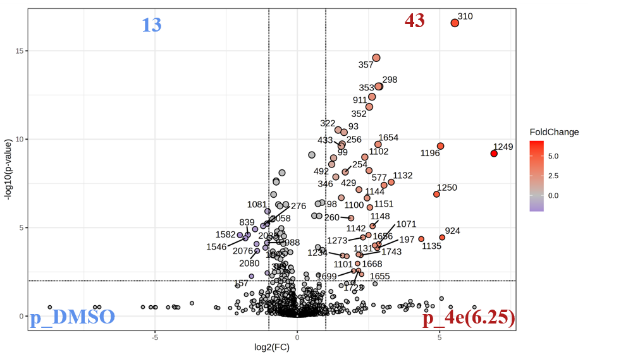
**

**Fig. S7. Metabolomics studies in cell pellet (C18 column).** **(A).** Heat map of the top 25 features obtained from pellet with highest scores from the PLS-DA and volcano plot **(B)** showing the number of differentially expressed features between 4e-treated groups (31.25 (μg/mL)) when compared to DMSO-untreated group. **(C).** Heat map of the top 25 features obtained from pellet with highest scores from the PLS-DA and volcano plot **(D)** showing the number of differentially expressed features between 4e-treated groups (6.25 (μg/mL)) when compared to DMSO-untreated group. Cut-off point of log2 fold-change ≤ -1.5, log2 fold-change ≥ 1.5; and false detection rate (FDR < 0.05) was used in this experiment.

**Figure S8**

**
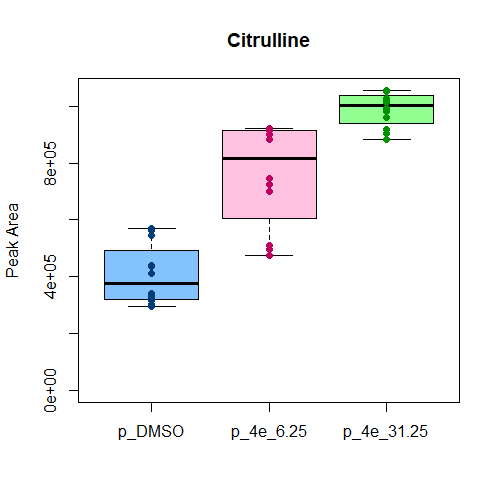

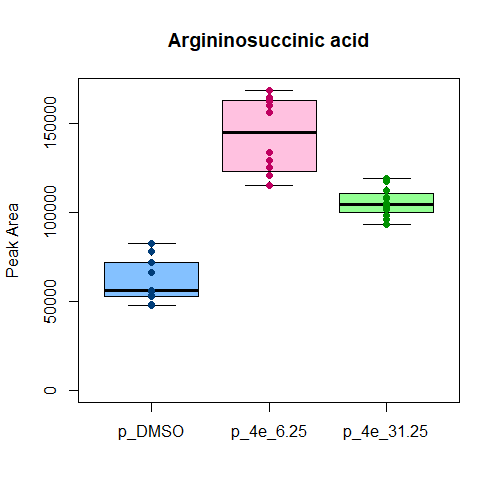

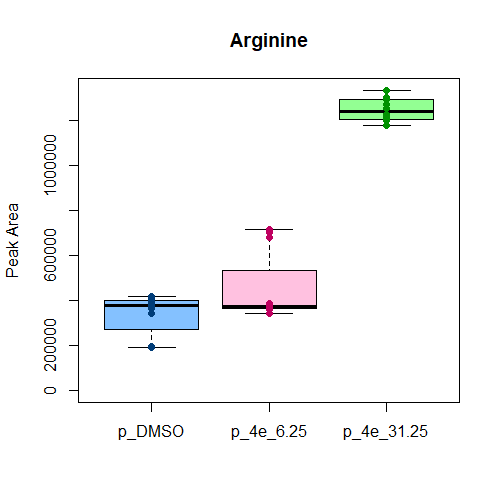
(A).**

**(B).**

**
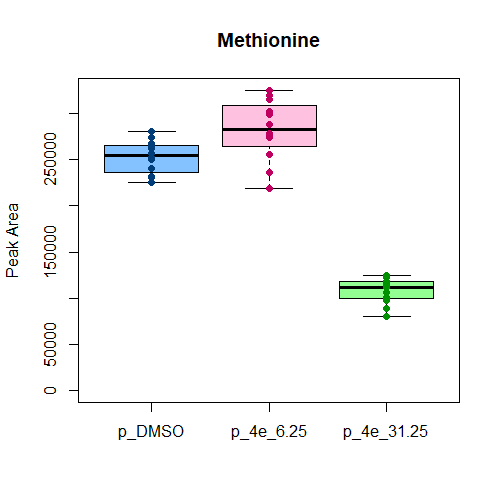

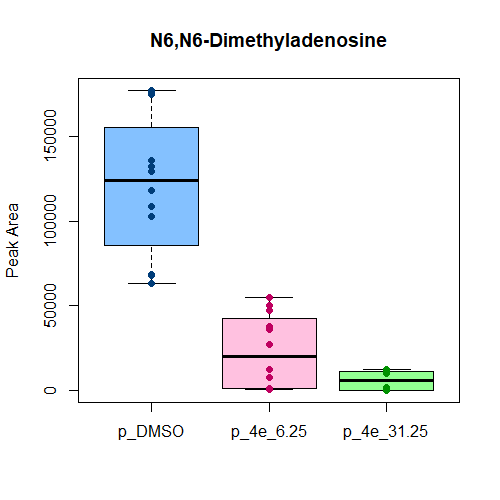
**

**Fig. S8. Upregulation of the metabolites in the urea cycle and maintenance of RNA stability.** **(A).** Urea cycle-associated metabolites. **(B).** mRNA stability-associated metabolites. Cut-off point of log2 fold-change ≤ -1.5, log2 fold-change ≥ 1.5; and false detection rate (FDR < 0.05) was used in this experiment.

**Figure S9**

**(A).**


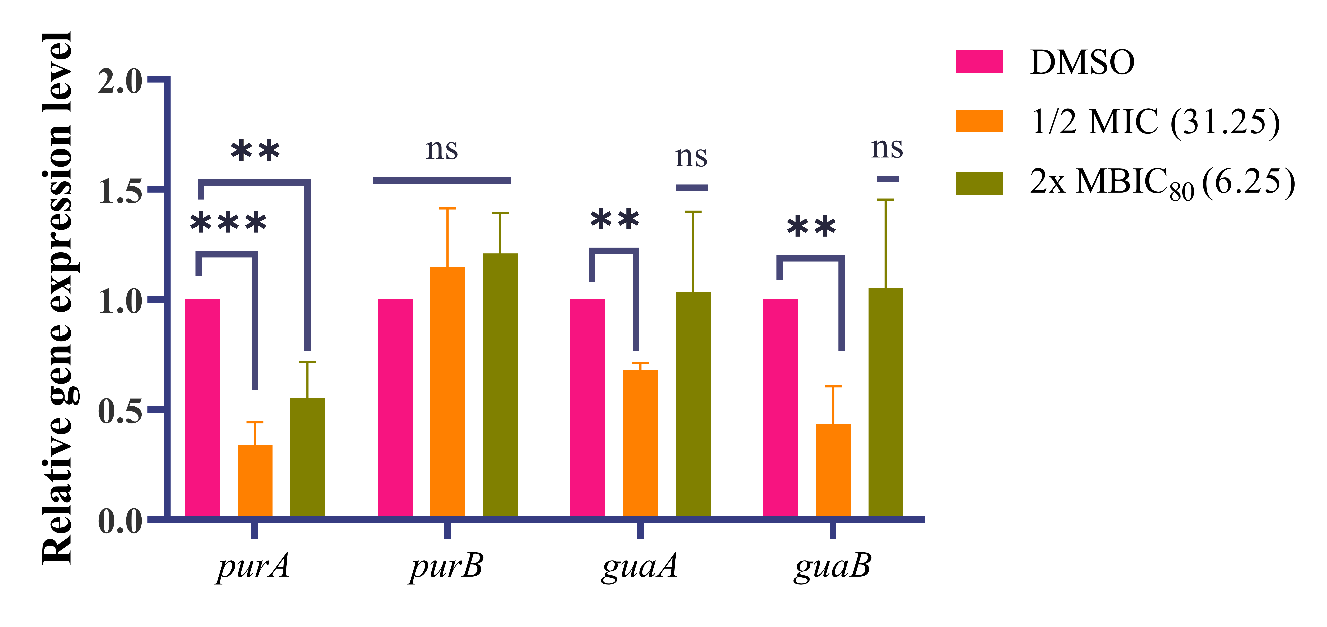


**(B).**


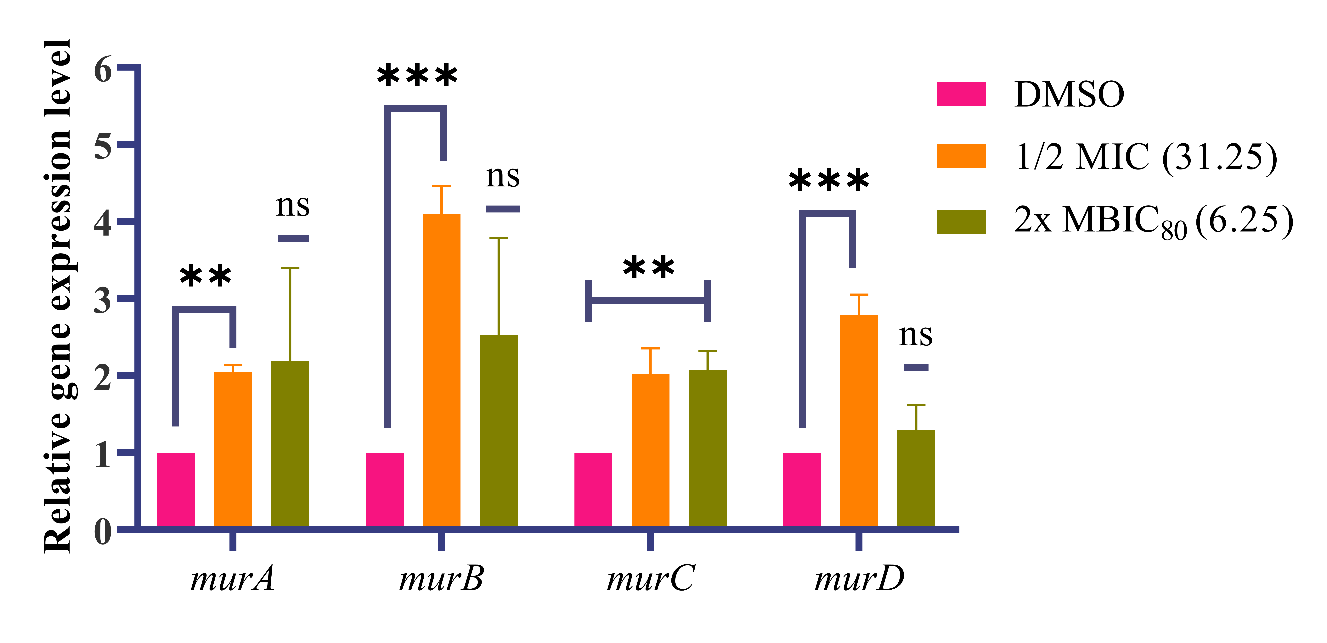


**Fig. S9. Gene expression determined using RT-qPCR.** **(A).** *De novo* purine biosynthesis associated genes. **(B).** Cell wall synthesis associated genes. A mid-exponential phase of CGMH-SL131 was treated with 1/2 MIC and 2x MBIC_80_ for 1 h and RNA was extracted. A 500 ng RNA was used for reverse transcription. The concentration of 4e (μg/mL) is indicated in parentheses. All experiments were done in three biological with three technical replicates. ns, no significance; **, *p*<0.01; ***, *p*<0.001.

**Figure S10**


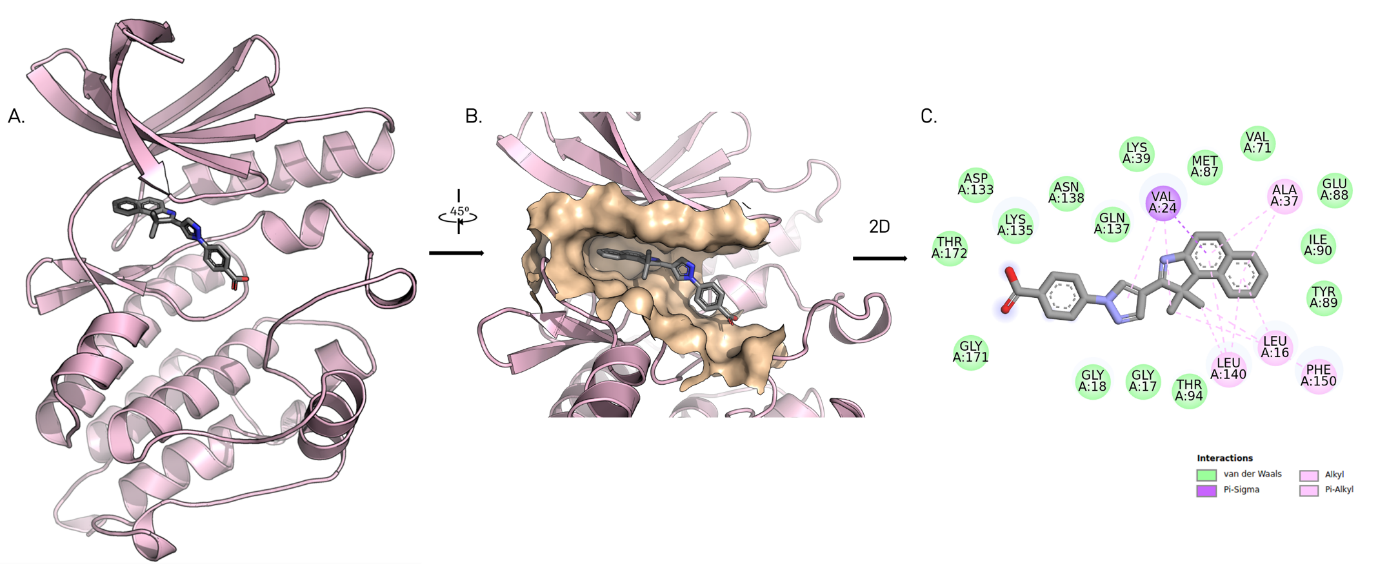


**Fig. S10. Binding mode and key interactions contributing to the compound's fit within the PASTA kinase active site**. The overall structure of the PASTA kinase in complex with compound 4e. The kinase is shown in pink cartoon representation, while compound 4e is depicted in stick representation and colored using the CPK color scheme (left). A close-up view of the binding site, with the binding pocket displayed in surface representation (middle). The figure demonstrates the precise fitting of compound 4e within the active site. The 2D interaction map of the PASTA kinase-compound 4e complex shows the specific interactions between the compound and the kinase (right). Molecular docking was performed using AutoDock Vina 1.5.6 to predict the binding pose and affinity of compound 4e. Visual inspection and image generation were done using Discovery Studio 2.5 and PyMOL.

**Figure S11**

**(A).**


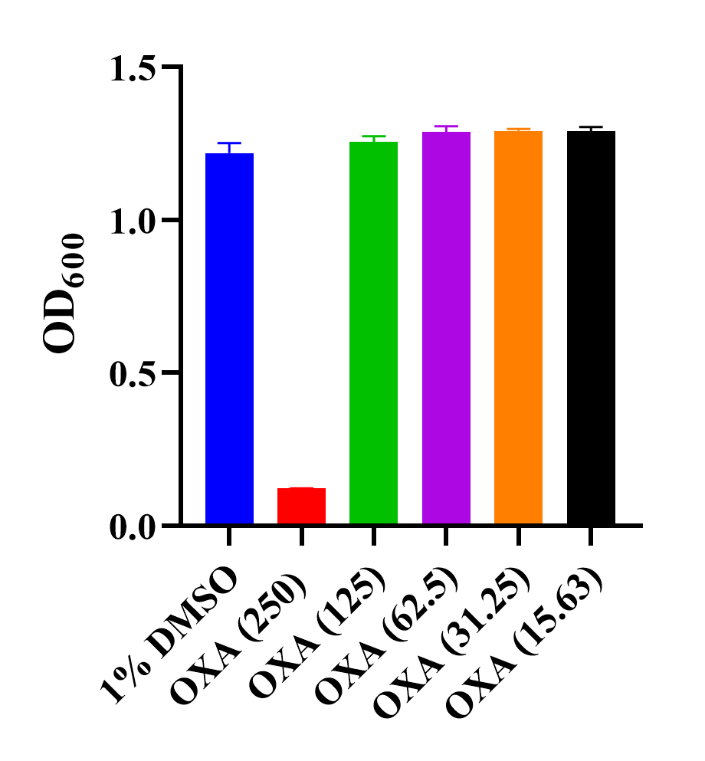


**(B).**

**Fig. S11. 4e enhanced oxacillin susceptibility of CGMH-SL131.** **(A).** MIC of CGMH-SL131 to oxacillin. **(B).** Oxacillin susceptibility in the presence of low dose of 4e (12.5 μg/mL). CGMH-SL131exposed to serially diluted concentration of oxacillin in the presence of 4e for 12 h. The concentration of 4e and oxacillin (μg/mL) is indicated in parentheses. All experiments were done in three biological with three technical replicates. Statistical analysis was performed using 1% DMSO-treated group as the comparison control. ***, *p*<0.001; ****, *p*<0.0001.
